# A Facile Ugi/Ullmann Cascade Reaction to Access Fused Indazolo-Quinoxaline Derivatives with Potent Anticancer Activity

**DOI:** 10.3390/molecules29020464

**Published:** 2024-01-17

**Authors:** Yong Li, Liujun He, Hongxia Qin, Yao Liu, Binxin Yang, Zhigang Xu, Donglin Yang

**Affiliations:** 1 College of Pharmacy, National & Local Joint Engineering Research Center of Targeted and Innovative Therapeutics, Chongqing University of Arts and Sciences, Chongqing 402160, China; 2 School of Chemistry and Chemical Engineering, Southwest University, Chongqing 400715, China

**Keywords:** heterocycles, multicomponent reactions (MCRs), organic synthesis, indazolo-fused quinoxalinone, anticancer activity

## Abstract

A facile methodology for the construction of a complex heterocycle indazolo-fused quinoxalinone has been developed via an Ugi four-component reaction (U-4CR) followed by an intramolecular Ullmann reaction. The expeditious process features an operationally simple approach, time efficiency, and a broad substrate scope. Biological activity was evaluated and demonstrated that compound **6e** inhibits human colon cancer cell HCT116 proliferation with an IC_50_ of 2.1 μM, suggesting potential applications for developing a drug lead in medicinal chemistry.

## 1. Introduction

**Figure 1:**
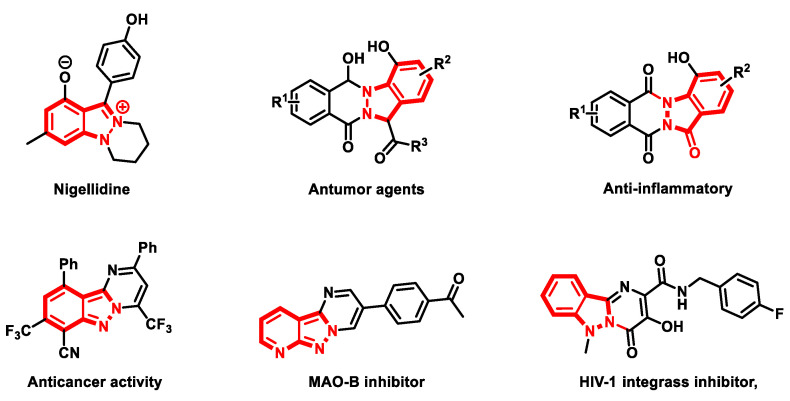
Natural product, bioactive, and drug molecules containing indazole structure.

Fused nitrogen polycyclic aromatic systems are one of the privileged core skeletons that are of great significance in biochemistry and the pharmaceutical industry [1,2,3,4]. Indazole and quinoxalinone cores, as two of them, are not only key scaffolds of many natural products but also essential units of a number of synthetic compounds possessing a wide spectrum of pharmaceutical activities [5,6,7,8,9,10,11,12,13,14,15]. In addition, indazole-fused polycyclic conjugated skeletons have received considerable interest as they are frequently encountered in natural products, biologically active compounds, and fluorescent probes (Figure 1) [16,17,18,19,20,21].

**Scheme 1:**
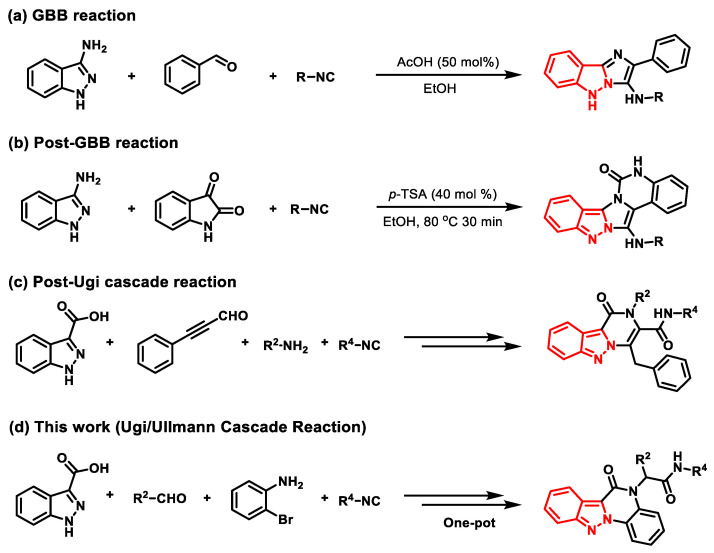
Strategies for the synthesis of indazolo-fused polycyclics via isocyanide-based multicomponent reactions.

Despite their importance, reliable methods for the synthesis of indazole-related polyheterocycles (PHCs) are rather limited [22,23,24,25], and some of them still suffer from the use of highly functionalized substrates, the production of a large amount of waste, and low atom economy. In recent years, isocyanide-based multicomponent reactions have been reported for the construction of indazole-related polyheterocycles with a facile and efficient process. For example, Jeong and coworkers reported the synthesis of novel 2-aryl-5*H*-imidazo [1,2-*b*]indazol-3-amine derivatives by using the Groebke–Blackburn–Bienaymé (GBB) reaction, which involved the reagents of 3-amino-1*H* indazoles, aldehydes, and isonitriles (Scheme 1a) [26]. Then, the same team reported the synthesis of novel amino-indazolo[3′,2′:2,3]imidazo[1,5-*c*]quinazolin-6(5*H*)-ones via a sequential post-GBB cyclization/spiro ring expansion triggered by a [1,5]-hydride shift (Scheme 1b) [27]. Our group has also reported the synthesis of pyrazole-pyrazines using a catalyst-free post-Ugi cascade reaction (Scheme 1c) [28]. In spite of this, more efficient synthesis methods for the construction of complex polyheterocycles are still desired.

As an evergreen in organic chemistry, multicomponent reactions (MCRs) never became old-fashioned or tedious [29,30,31,32,33], because they always inspire creative spirits by following the fundamental quest: more than two compounds are reacted in a one-pot fashion to form two or more bonds. The post-Ugi reactions [34,35,36,37], as typical MCRs, are well suited for the construction of important heterocycles, macrocycles, polymers, and other compounds in drug discovery and natural product synthesis [38,39,40,41,42,43]. In the course of our continued study on the construction of novel *N*-fused heterocyclic chemical spaces and activity evaluation from Ugi adductive [28,36,44,45,46], we hoped to establish a highly efficient diversity-oriented synthetic route to indazole-fused polycyclics utilizing a sequential one-pot Ugi/Ullmann reaction, as shown in Scheme 1d. We reasoned that the Ugi product involving 3-carboxyindazole and 2-bromoaniline would undergo an intramolecular Ullmann cyclization cascade process to yield the indazolo-quinoxaline derivatives. As excepted, indazolo-quinoxaline derivatives were synthesized by a facile and efficient process and are discussed below.

## 2. Results and Discussion

The starting materials, 3-carboxyindazole **1a**, 4-chlorobenzaldehyde **2a**, 2-bromoaniline **3**, and *tert*-butyl isocyanide **4a**, were mixed in methanol at room temperature and under air to afford the desired Ugi product **5a**. With compound **5a** in hand, we proceeded to optimize the conditions for the subsequent Ullmann reaction, and the results are shown in Table 1. Compound **5a** was treated in *N*,*N*-dimethylformamide (DMF) with different bases under a variety of microwave irradiation conditions. When an inorganic base was used, the target compound **6a** was observed in a relatively low yield (55%) (Table 1, entry 1–5). The results of these screening experiments revealed that when organic bases 1,8-diazabicyclo[5.4.0]undec-7-ene (DBU), 4-dimethylaminopyridine (DMAP), and triethylamine) (TEA) were used, target compound **6a** was observed in the LC/MS spectrum with very low yield (Table 1, entries 6–8). Addition of a copper catalyst and ligand resulted in a considerable increase in the yield (Table 1, entries 9–17). Different ligands were screened by performing the reaction with K_2_CO_3_ and CuI (Table 1, entries 9–14), and the results showed that when tetramethylethylenediamine (TMEDA) was used as the ligand, compound **6** was obtained with a high yield (95%). When we carried out the reaction using another copper catalyst, such as CuBr, Cu_2_O, and Cu(OTf)_2_, in the presence of TMEDA, we only achieved yields of 49–65% (entries 15–17). The results proved that the optimal conditions for the Ullmann reaction were K_2_CO_3_ as the base in the presence of CuI and TMEDA in DMF under microwave at 150 °C for 30 min. After the Ullmann reaction conditions were optimized, we continued to investigate the possibility of carrying out this reaction as a one-pot procedure.

**Scheme 2:**
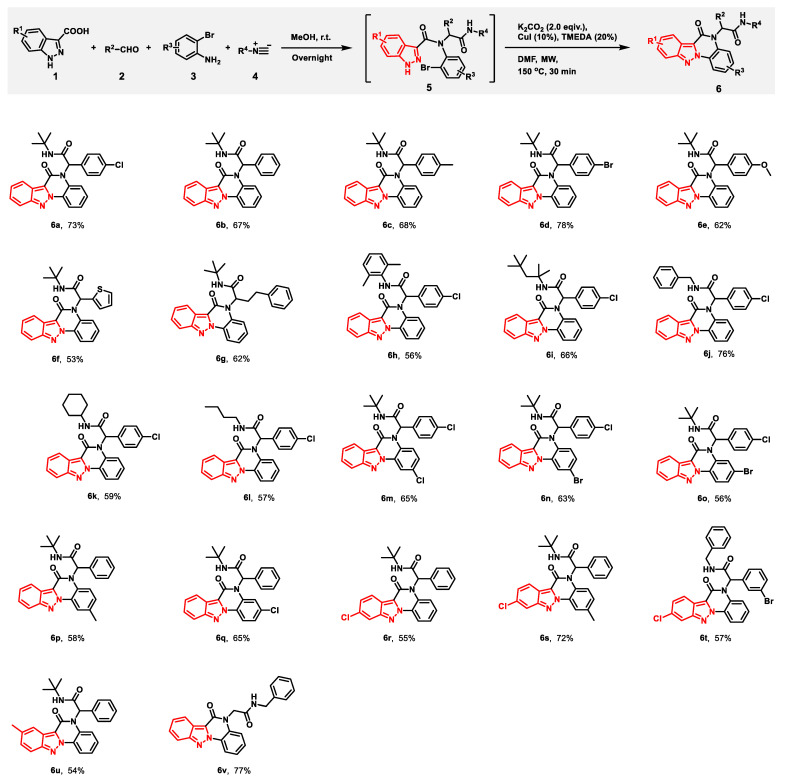
Scope of the Ugi/Ullmann reaction route leading to indazolo-quinoxaline derivatives **6a**–**6v** ^a^. ^a^ Reaction conditions: **1** (1.0 mmol), **2** (1.0 mmol), **3** (1.0 mmol), and **4** (1.0 mmol) in MeOH (2.0 mL) under air. After the solvent removed, added K_2_CO_3_ (2.0 mmol), CuI (0.1 mmol), and TMEDA (0.2 mmol) in DMF (5.0 mL) under microwave irradiation at 150 °C for 30 min. Overall yield of isolated product.

With the optimized intramolecular Ullmann cyclization reaction conditions in hand, we investigated the cyclization reaction with the unpurified Ugi product **5a**. In all cases, the initial Ugi products were used directly in the next step without further purification after the removal of the solvent. In one pot, the cascade reaction produced the final product **6a** with a 73% yield for two steps, which showed no discernible impact on the overall yield. We then investigated the scope of this one-pot procedure by varying the starting materials (in Scheme 2). First, different aldehydes were tested. The results showed that different substituents on the aryl aldehydes exhibited good performance, affording compounds **6a**–**6e** with 62–78% yield, and the electronic properties of the substituent did not impact the reactivity. Strikingly, when heterocyclic aldehydes and aliphatic aldehydes were used as input in the Ugi reaction, compounds **6f** and **6g** were also obtained, with 53% and 62% yields, respectively. Next, different isocyanides were used as Ugi inputs, and the results showed that the corresponding indazolo-quinoxalines **6i**–**6h** were generated smoothly with a 56–76% yield. The substituents of the benzene of aniline were also investigated for obtaining compounds **6m**–**6q**. Further investigations of the substituents on carboxyindazole revealed that different substituents didn’t make a significant difference in the reaction yield. The Cl- or Me-substituted carboxyindazoles were involved in the protocol, and the products **6r**–**6u** were obtained in 76–85% yields. It is worth noting that when formaldehyde was used, the product **6v** was obtained with a 77% yield. All the results showed that a variety of different starting materials were successfully employed under the optimized conditions for the construction of structurally diverse indazolo-quinoxalines **6a**–**6v,** with yields in the range of 53–78% (Scheme 2), indicating a good functional group tolerance.

**Figure 2:**
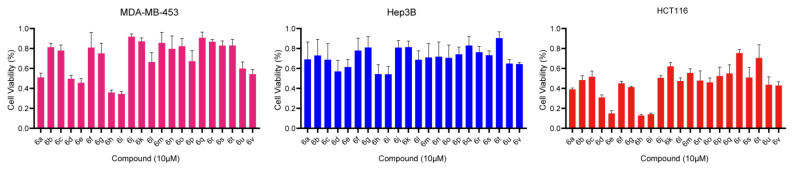
Anticancer activities of compounds **6a**–**v** against different cancer cell lines. All MTT assays were repeated three times using six samples per assay. Proliferation inhibition activity of compounds **6a**–**v** was assessed by MTT in breast cancer cells (MDA-MB-453), liver cancer cells (Hep3B), and colon cells (HCT116). These cells were treated with compounds **6** at 10 μM for 48 h, respectively, then cell viability was measured.

**Figure 3:**
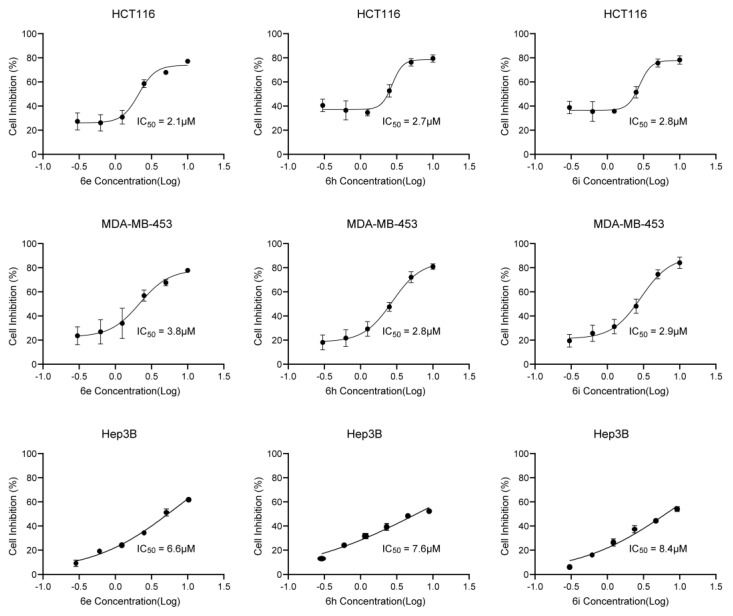
The half-maximal inhibitory concentration (IC_50_) of compound **6e**, **6h**, and **6i** was determined in HCT116, MDA-MB-453, and Hep3B cell lines.

To extend the application of the synthesized compound in medical chemistry for developing a drug lead from compounds **6**, the antiproliferative effect of compounds 6 was evaluated in several human cancer cell lines. To our delight, a significant cell growth inhibitory effect was observed, especially compound **6e**, which exhibited better anticancer activities in the human colorectal cancer HCT116 cell lines than other cell lines (Figure 2). It is worth noting that compounds **6e**, **6h**, and **6i** showed excellent anticancer activities, with IC_50_ of 2.1 μM, 2.7 μM, and 2.8 μM, respectively (Figure 3), which is comparable to 36.80 for etoposide, as reported in our previous work [44]. Further efforts are ongoing to explore the mechanism of action of drug leads and optimize their potency and drug properties.

## 3. Materials and Methods

### 3.1. General Information

^1^H and ^13^C NMR data were recorded on a Bruker (Billerica, MA, USA) 400 spectrometer. ^1^H NMR data are reported as follows: chemical shift in ppm (δ), multiplicity (s = singlet, d = doublet, t = triplet, m = multiplet), coupling constant (Hz), relative intensity. ^13^C NMR data are reported as follows: chemical shift in ppm (δ). HPLC-MS analyses were performed on a Shimadzu-2020 LC-MS instrument (Kyoto City, Japan) using the following conditions: Shim-pack VPODS C18 column (reverse phase, 150 × 2.0 mm); 80% acetonitrile and 20% water over 6.0 min; flow rate of 0.4 mL/min; UV photodiode array detection from 200 to 300 nm. The products were purified using Biotage (Uppsala, Sweden) Isolera™ Spektra Systems and hexane/EtOAc solvent systems. Unless otherwise specified, all chemicals were purchased from commercial sources and used without further treatment. All microwave irradiation experiments were carried out according to our previous report [44,45].

### 3.2. Cell Lines and Culture

The human tumor cells Hep3B, HCT116, and MDA-MB-453 were purchased from the American Type Culture Collection (ATCC, Manassas, VA, USA). Hep3B cells were cultured with MEM (Pricella, PM150410, Wuhan, China) and supplemented with 10% fetal bovine serum (FBS, Gibco, 10100147, Waltham, MA, USA, Australian origin) and 1 mM sodium pyruvate (NaP). HCT116 cells were cultured in McCoy’s 5a medium (Gibco, 16600108, Waltham, MA, USA) supplemented with 10% FBS. MDA-MB-453 cells were cultured with high-glucose DMEM medium (Hyclone, SH30022.01, Logan, UT, USA) with 10% FBS added. All used cells were incubated in an incubator at 37 °C and 5% CO_2_ in a humidified atmosphere.

### 3.3. Cell Viability Assay

The cell viability and the IC_50_ value of compounds **6a**–**v** in the human tumor cells were evaluated using a 3-(4,5-dimethyl-2-thiazolyl)-2,5-diphenyl-2-*H*-tetrazolium bromide (MTT, Beyotime, ST316, Shanghai, China) assay. Briefly, the tumor cells were harvested, counted, and seeded into the 96-well plate, with a density of 2 × 10^3^ cells per well. After incubation for 24 h, 100 µL of medium with 20 µM compounds were added and incubated for 2 days for the initial screening. To assess anticancer activity, 20 μL of MTT solution (5 mg/mL in PBS) were added to each well and incubated for another 4 h. Then, the medium was removed, and 200 μL of DMSO was added to each well to dissolve the formazan cystals. The absorbance was measured at 570 nm using a microplate reader (Bio-Tek, Winooski, VT, USA). The experiments were repeated three times, and growth inhibition curves and IC_50_ values were generated using GraphPad Prism 8.0.

### 3.4. General Procedure for Synthesis of Compounds ***6a**–**v***

Aldehyde (1.0 mmol, 1.0 eq.), MeOH (2 mL), and amine (1.0 mmol, 1.0 eq.) were sequentially added to a 10 mL microwave vial and stirred for 10 min. under air. Then, carboxylic acid (1.0 mmol, 1.0 eq.) and isocyanide (1.0 mmol, 1.0 eq.) were added separately. The mixture was stirred for 6–12 h at room temperature and monitored with TLC. When no isocyanide was present, the solvent was removed using rotary evaporation. The residue was dissolved in DMF (5 mL) in a 10 mL microwave vial, then, K_2_CO_3_ (2.0 eq.), CuI (20 mol%), and TMEDA (20 mol%) were added. The vial was sealed and heated in a microwave at 150 °C for 30 min. The solvent was removed using rotary evaporation after the reaction was completed. The residue was dissolved in EtOAc (15 mL) and washed with brine. The EtOAc phase was dried over anhydrous Na_2_SO_4_ and concentrated. The residue was purified using silica gel column chromatography using a gradient of EtOAc/hexane (0–100%) to afford the relative product **6**.

*N*-(*tert*-butyl)-2-(4-chlorophenyl)-2-(6-oxoindazolo[2,3-*a*]quinoxalin-5(6*H*)-yl)acetamide (**6a**), purified by flash chromatography using a gradient of EtOAc/hexane (0–40%), white solid, 289 mg, yield 62%. ^1^H NMR (400 MHz, CDCl_3_) δ 8.68 (dd, *J* = 7.8, 1.8 Hz, 1H), 8.38 (d, *J* = 8.4 Hz, 1H), 8.00 (d, *J* = 8.7 Hz, 1H), 7.61–7.49 (m, 2H), 7.47–7.36 (m, 3H), 7.32 (q, *J* = 8.9 Hz, 4H), 6.98 (s, 1H), 5.99 (s, 1H), 1.33 (s, 9H). ^13^C NMR (101 MHz, CDCl_3_) δ 166.50, 155.64, 149.05, 134.04, 132.56, 129.35, 129.27, 128.94, 128.30, 128.20, 125.23, 125.16, 124.51, 122.70, 121.85, 121.00, 118.47, 117.84, 117.73, 59.10, 52.24, 28.56. HRMS (ESI) *m*/*z* calcd. for C_26_H_24_ClN_4_O_2_^+^ (M + H)^+^ 459.1582, found 459.1580.

*N*-(*tert*-butyl)-2-(6-oxoindazolo[2,3-*a*]quinoxalin-5(6*H*)-yl)-2-phenylacetamide (**6b**), ^1^H NMR (400 MHz, CDCl_3_) δ 8.66 (dd, *J* = 7.8, 1.9 Hz, 1H), 8.37 (d, *J* = 8.4 Hz, 1H), 7.98 (d, *J* = 8.7 Hz, 1H), 7.60–7.51 (m, 2H), 7.46–7.27 (m, 8H), 6.94 (s, 1H), 6.02 (s, 1H), 1.35 (s, 9H). ^13^C NMR (101 MHz, CDCl_3_) δ 166.70, 155.70, 149.15, 134.16, 129.87, 128.97, 128.27, 128.09, 127.83, 125.17, 125.09, 124.28, 123.04, 121.94, 121.35 118.35, 117.79, 117.65, 60.54, 52.12, 28.57. HRMS (ESI) *m*/*z* calcd. for C_26_H_25_N_4_O_2_^+^ (M + H)^+^ 425.1972, found 425.1974.

*N*-(*tert*-butyl)-2-(6-oxoindazolo[2,3-*a*]quinoxalin-5(6*H*)-yl)-2-(*p*-tolyl)acetamide (**6c**), ^1^H NMR (400 MHz, CDCl_3_) δ 8.66 (d, *J* = 9.7 Hz, 1H), 8.39 (d, *J* = 8.4 Hz, 1H), 8.03–7.93 (m, 1H), 7.60–7.51 (m, 2H), 7.45–7.33 (m, 3H), 7.27–7.19 (m, 3H), 7.13 (d, *J* = 7.1 Hz, 1H), 6.85 (s, 1H), 5.98 (s, 1H), 2.31 (s, 3H), 1.35 (s, 9H). ^13^C NMR (101 MHz, CDCl_3_) δ 166.74, 155.69, 149.16, 138.87, 134.16, 130.02, 129.15, 128.88, 128.44, 128.09, 125.17, 125.05, 124.86, 124.22, 123.13, 121.94, 121.42, 118.26, 117.77, 60.81, 52.08, 28.58, 21.58. HRMS (ESI) *m*/*z* calcd. for C_27_H_27_N_4_O_2_^+^ (M + H)^+^ 439.2129, found 439.2129.

2-(4-Bromophenyl)-*N*-(*tert*-butyl)-2-(6-oxoindazolo[2,3-*a*]quinoxalin-5(6*H*)-yl)acetamide (**6d**), ^1^H NMR (400 MHz, CDCl_3_) δ 8.67 (dd, *J* = 8.0, 1.7 Hz, 1H), 8.35 (d, *J* = 8.4 Hz, 1H), 7.99 (d, *J* = 8.7 Hz, 1H), 7.60–7.50 (m, 2H), 7.49–7.34 (m, 5H), 7.28 (s, 1H), 7.26 (s, 1H), 6.97 (s, 1H), 6.03 (s, 1H), 1.32 (s, 9H). ^13^C NMR (101 MHz, CDCl_3_) δ 166.33, 155.64, 149.18, 132.99, 131.92, 129.54, 129.33, 128.39, 128.25, 125.33, 125.20, 124.56, 122.79, 122.24, 121.99, 121.22, 118.39, 117.89, 117.78, 59.19, 52.24, 28.56. HRMS (ESI) *m*/*z* calcd. for C_26_H_24_BrN_4_O_2_^+^ (M + H)^+^ 503.1077, found 503.1075.

*N*-(*tert*-butyl)-2-(4-methoxyphenyl)-2-(6-oxoindazolo[2,3-*a*]quinoxalin-5(6*H*)-yl)acetamide (**6e**), ^1^H NMR (400 MHz, CDCl_3_) δ 8.68–8.59 (m, 1H), 8.38 (d, *J* = 8.4 Hz, 1H), 7.97 (d, *J* = 8.7 Hz, 1H), 7.62–7.49 (m, 2H), 7.44–7.32 (m, 5H), 6.88 (d, *J* = 8.8 Hz, 2H), 6.81 (s, 1H), 5.96 (s, 1H), 3.78 (s, 3H), 1.35 (s, 9H). ^13^C NMR (101 MHz, CDCl_3_) δ 166.99, 159.43, 155.65, 149.13, 129.98, 129.36, 128.24, 128.08, 126.10, 125.17, 125.01, 124.19, 123.13, 121.90, 121.39, 118.10, 117.75, 117.66, 114.39, 60.37, 55.31, 52.05, 28.59. HRMS (ESI) *m*/*z* calcd. for C_27_H_27_N_4_O_3_^+^ (M + H)^+^ 455.2078, found 455.2086.

*N*-(*tert*-butyl)-2-(6-oxoindazolo[2,3-*a*]quinoxalin-5(6*H*)-yl)-2-(thiophen-2-yl)acetamide (**6f**) ^1^H NMR (400 MHz, CDCl_3_) δ 8.69–8.62 (m, 1H), 8.39 (d, *J* = 8.4 Hz, 1H), 7.97 (d, *J* = 8.7 Hz, 1H), 7.56 (ddd, *J* = 8.8, 4.9, 1.6 Hz, 2H), 7.46–7.37 (m, 3H), 7.32 (dd, *J* = 5.1, 1.1 Hz, 1H), 7.09 (d, *J* = 3.0 Hz, 1H), 6.94 (dd, *J* = 5.1, 3.7 Hz, 1H), 5.95 (s, 1H), 5.29 (s, 1H),1.33 (s, 9H). ^13^C NMR (101 MHz, CDCl_3_) δ 166.23, 155.20, 149.14, 135.75, 128.34, 128.09, 127.38, 126.49, 125.26, 124.44, 122.00, 121.28, 117.85, 53.45, 52.25, 28.52. HRMS (ESI) *m*/*z* calcd. for C_24_H_23_N_4_O_2_S^+^ (M + H)^+^ 431.1536, found 431.1539.

*N*-(*tert*-butyl)-2-(6-oxoindazolo[2,3-*a*]quinoxalin-5(6*H*)-yl)-4-phenylbutanamide (**6g**), ^1^H NMR (400 MHz, CDCl_3_) δ 8.58 (dd, *J* = 8.1, 1.3 Hz, 1H), 8.30 (d, *J* = 8.4 Hz, 1H), 7.95 (d, *J* = 8.7 Hz, 1H), 7.65 (d, *J* = 7.9 Hz, 1H), 7.58–7.52 (m, 1H), 7.51–7.46 (m, 1H), 7.45–7.36 (m, 2H), 6.94 (s, 5H), 6.27 (s, 1H), 5.87 (s, 1H), 2.80 (ddd, *J* = 21.6, 13.4, 7.2 Hz, 2H), 2.48 (dd, *J* = 11.1, 6.1 Hz, 2H), 1.28 (s, 9H). ^13^C NMR (101 MHz, CDCl_3_) δ 168.93, 155.94, 149.01, 140.11, 128.29, 128.02, 127.99, 125.83, 125.22, 125.16, 124.42, 121.79, 121.04, 117.94, 117.80, 55.21, 51.82, 33.01, 28.55. HRMS (ESI) *m*/*z* calcd. for C_28_H_29_N_4_O_2_^+^ (M + H)^+^ 453.2285, found 453.2286.

2-(4-Chlorophenyl)-*N*-(2,6-dimethylphenyl)-2-(6-oxoindazolo[2,3-*a*]quinoxalin-5(6*H*)-yl)acetamide (**6h**), ^1^H NMR (400 MHz, CDCl_3_) δ 8.67 (d, *J* = 8.0 Hz, 1H), 8.33 (d, *J* = 8.3 Hz, 1H), 7.98 (d, *J* = 8.7 Hz, 1H), 7.68–7.52 (m, 3H), 7.49 (d, *J* = 8.4 Hz, 2H), 7.40 (ddd, *J* = 20.5, 10.5, 7.2 Hz, 5H), 7.22 (s, 1H), 7.04 (dt, *J* = 17.4, 6.6 Hz, 3H), 2.13 (s, 6H). ^13^C NMR (101 MHz, CDCl_3_) δ 165.72, 155.76, 149.19, 135.16, 134.41, 133.05, 132.14, 129.31, 128.47, 128.34, 127.64, 125.46, 125.31, 124.78, 122.69, 121.99, 121.13, 118.24, 117.96, 58.84, 18.47. HRMS (ESI) *m*/*z* calcd. for C_30_H_24_ClN_4_O_2_^+^ (M + H)^+^ 507.1582, found 507.1572.

2-(4-Chlorophenyl)-2-(6-oxoindazolo[2,3-*a*]quinoxalin-5(6*H*)-yl)-*N*-(2,4,4-trimethylpentan-2-yl)acetamide (**6i**), ^1^H NMR (400 MHz, CDCl_3_) δ 8.65 (d, *J* = 7.6 Hz, 1H), 8.34 (d, *J* = 8.4 Hz, 1H), 7.97 (d, *J* = 8.7 Hz, 1H), 7.55 (dd, *J* = 15.5, 7.6 Hz, 2H), 7.36 (ddd, *J* = 26.0, 16.2, 8.0 Hz, 7H), 6.97 (s, 1H), 6.11 (s, 1H), 1.64 (q, *J* = 15.0 Hz, 2H), 1.39 (d, *J* = 13.2 Hz, 6H), 0.80 (s, 9H). ^13^C NMR (101 MHz, CDCl_3_) δ 165.98, 155.55, 149.14, 134.04, 132.33, 129.36, 128.91, 128.36, 128.29, 125.28, 125.16, 124.56, 122.71, 121.93, 121.19, 118.37, 117.89, 117.77, 59.13, 56.19, 52.45, 31.50, 31.20, 28.85, 28.32. HRMS (ESI) *m*/*z* calcd. for C_30_H_32_ClN_4_O_2_^+^ (M + H)^+^ 515.2208, found 515.2210.

*N*-benzyl-2-(4-chlorophenyl)-2-(6-oxoindazolo[2,3-*a*]quinoxalin-5(6*H*)-yl)acetamide (**6j**) ^1^H NMR (400 MHz, CDCl_3_) δ 8.57 (dd, *J* = 7.9, 1.8 Hz, 1H), 8.10 (d, *J* = 8.4 Hz, 1H), 7.92 (d, *J* = 8.7 Hz, 1H), 7.55–7.45 (m, 2H), 7.41–7.31 (m, 4H), 7.31–7.26 (m, 3H), 7.21–7.11 (m, 5H), 7.04 (s, 1H), 6.84 (t, *J* = 5.3 Hz, 1H), 4.55–4.42 (m, 2H). ^13^C NMR (101 MHz, CDCl_3_) δ 167.28, 155.65, 149.01, 137.50, 134.25, 132.08, 129.34, 129.18, 129.04, 128.61, 128.33, 127.72, 127.53, 125.32, 125.22, 124.58, 122.59, 121.79, 120.88, 118.13, 117.84, 117.80, 58.38, 44.08. HRMS (ESI) *m*/*z* calcd. for C_29_H_22_ClN_4_O_2_^+^ (M + H)^+^ 493.1426, found 493.1419.

2-(4-Chlorophenyl)-*N*-cyclohexyl-2-(6-oxoindazolo[2,3-*a*]quinoxalin-5(6*H*)-yl)acetamide (**6k**), ^1^H NMR (400 MHz, CDCl_3_) δ 8.61 (d, *J* = 8.0 Hz, 1H), 8.21 (d, *J* = 8.3 Hz, 1H), 7.93 (d, *J* = 8.7 Hz, 1H), 7.57–7.48 (m, 2H), 7.42–7.25 (m, 7H), 7.03 (s, 1H), 6.36 (d, *J* = 7.6 Hz, 1H), 3.91–3.73 (m, 1H), 1.95 (d, *J* = 10.6 Hz, 1H), 1.72 (d, *J* = 11.9 Hz, 1H), 1.59 (dd, *J* = 35.6, 7.8 Hz, 3H), 1.38–1.20 (m, 2H), 1.19–1.09 (m, 1H), 1.01 (ddd, *J* = 14.9, 14.3, 4.2 Hz, 2H). ^13^C NMR (101 MHz, CDCl_3_) δ 166.32, 155.60, 149.03, 134.10, 132.40, 129.30, 128.96, 128.32, 128.25, 125.25, 125.16, 124.53, 122.67, 121.80, 121.00, 118.29, 117.84, 117.75, 58.58, 49.16, 32.69, 32.60, 25.34, 24.82, 24.75. HRMS (ESI) *m*/*z* calcd. for C_28_H_26_ClN_4_O_2_^+^ (M + H)^+^ 485.1739, found 485.1725.

*N*-butyl-2-(4-chlorophenyl)-2-(6-oxoindazolo[2,3-*a*]quinoxalin-5(6*H*)-yl)acetamide (**6l**), ^1^H NMR (400 MHz, CDCl_3_) δ 8.57 (d, *J* = 7.2 Hz, 1H), 8.16 (d, *J* = 8.3 Hz, 1H), 7.92 (d, *J* = 8.7 Hz, 1H), 7.51 (dd, *J* = 14.9, 7.5 Hz, 2H), 7.40–7.26 (m, 7H), 7.01 (s, 1H), 6.57 (s, 1H), 3.23 (dd, *J* = 13.3, 6.6 Hz, 2H), 1.46–1.35 (m, 2H), 1.28–1.13 (m, 2H), 0.80 (t, *J* = 7.3 Hz, 3H). ^13^C NMR (101 MHz, CDCl_3_) δ 167.23, 155.63, 148.99, 134.13, 132.30, 129.33, 128.97, 128.32, 125.25, 125.16, 124.53, 122.62, 121.73, 120.88, 118.19, 117.84, 117.74, 58.45, 39.88, 31.27, 19.99, 13.66. HRMS (ESI) *m*/*z* calcd. for C_26_H_24_ClN_4_O_2_^+^ (M + H)^+^ 459.1582, found 459.1571.

*N*-(*tert*-butyl)-2-(2-chloro-6-oxoindazolo[2,3-*a*]quinoxalin-5(6*H*)-yl)-2-(4-chlorophenyl)acetamide (**6m**), ^1^H NMR (400 MHz, CDCl_3_) δ 8.63 (d, *J* = 2.1 Hz, 1H), 8.28 (d, *J* = 8.4 Hz, 1H), 7.95 (d, *J* = 8.7 Hz, 1H), 7.59–7.53 (m, 1H), 7.50 (d, *J* = 9.1 Hz, 1H), 7.44–7.38 (m, 1H), 7.34–7.25 (m, 5H), 7.02 (s, 1H), 6.15 (s, 1H), 1.34 (s, 9H). ^13^C NMR (101 MHz, CDCl_3_) δ 166.21, 155.40, 149.31, 134.25, 132.27, 130.30, 129.11, 129.08, 128.71, 128.12, 127.80, 125.78, 125.64, 122.76, 122.01, 121.11, 120.06, 117.97, 117.57, 58.86, 52.37, 28.59. HRMS (ESI) *m*/*z* calcd. for C_26_H_23_Cl_2_N_4_O_2_^+^ (M + H)^+^ 493.1193, found 493.1186.

2-(2-Bromo-6-oxoindazolo[2,3-*a*]quinoxalin-5(6*H*)-yl)-*N*-(*tert*-butyl)-2-(4-chlorophenyl)acetamide (**6n**), ^1^H NMR (400 MHz, CDCl_3_) δ 8.73 (d, *J* = 1.2 Hz, 1H), 8.16 (d, *J* = 8.4 Hz, 1H), 7.90 (d, *J* = 8.7 Hz, 1H), 7.55–7.49 (m, 1H), 7.44 (dd, *J* = 13.4, 5.2 Hz, 2H), 7.38–7.25 (m, 5H), 7.04 (s, 1H), 6.32 (s, 1H), 1.34 (s, 9H). ^13^C NMR (101 MHz, CDCl_3_) δ 166.24, 155.39, 149.20, 136.73, 134.23, 132.33, 130.94, 129.09, 128.66, 128.27, 125.93, 125.59, 122.67, 121.90, 120.94, 120.42, 120.34, 117.92, 117.52, 87.50, 58.84, 52.38, 28.60. HRMS (ESI) *m*/*z* calcd. for C_26_H_23_BrClN_4_O_2_^+^ (M + H)^+^ 537.0687, found 537.0678.

2-(3-Bromo-6-oxoindazolo[2,3-*a*]quinoxalin-5(6*H*)-yl)-*N*-(tert-butyl)-2-(4-chlorophenyl)acetamide (**6o**), ^1^H NMR (400 MHz, CDCl_3_) δ 8.51 (d, *J* = 8.7 Hz, 1H), 8.34 (d, *J* = 8.4 Hz, 1H), 7.96 (d, *J* = 8.7 Hz, 1H), 7.67 (s, 1H), 7.59–7.54 (m, 1H), 7.51 (d, *J* = 8.8 Hz, 1H), 7.46–7.41 (m, 1H), 7.37 (s, 4H), 6.83 (s, 1H), 5.91 (s, 1H), 1.37 (s, 9H). ^13^C NMR (101 MHz, CDCl_3_) δ 165.82, 155.33, 149.24, 134.62, 132.27, 130.59, 129.37, 128.59, 127.47, 125.54, 124.17, 122.68, 122.06, 121.78, 121.19, 120.76, 119.04, 117.88, 59.96, 52.38, 28.55. HRMS (ESI) *m*/*z* calcd. for C_26_H_23_BrClN_4_O_2_^+^ (M + H)^+^ 537.0687, found 537.0685.

*N*-(*tert*-butyl)-2-(2-methyl-6-oxoindazolo[2,3-*a*]quinoxalin-5(6*H*)-yl)-2-phenylacetamide (**6p**), ^1^H NMR (400 MHz, CDCl_3_) δ 8.47 (d, *J* = 1.0 Hz, 1H), 8.36 (dd, *J* = 8.4, 0.9 Hz, 1H), 7.97 (d, *J* = 8.7 Hz, 1H), 7.58–7.50 (m, 1H), 7.46–7.38 (m, 4H), 7.38–7.27 (m, 3H), 7.15 (dd, *J* = 8.7, 1.5 Hz, 1H), 6.97 (s, 1H), 6.06 (s, 1H), 2.47 (s, 3H), 1.34 (s, 9H). ^13^C NMR (101 MHz, CDCl_3_) δ 166.78, 155.60, 149.10, 134.61, 134.25, 129.13, 128.90, 128.24, 128.17, 127.81, 127.56, 124.97, 124.93, 123.10, 121.91, 121.37, 118.32, 117.68, 117.55, 60.21, 52.09, 28.59, 20.82. HRMS (ESI) *m*/*z* calcd. for C_27_H_27_N_4_O_2_^+^ (M + H)^+^ 439.2129, found 439.2127.

*N*-(*tert*-butyl)-2-(3-chloro-6-oxoindazolo[2,3-*a*]quinoxalin-5(6*H*)-yl)-2-phenylacetamide (**6q**), ^1^H NMR (400 MHz, CDCl_3_) δ 8.58 (dd, *J* = 8.8, 1.2 Hz, 1H), 8.37 (dd, *J* = 8.4, 1.1 Hz, 1H), 7.96 (d, *J* = 8.7 Hz, 1H), 7.60–7.50 (m, 2H), 7.46–7.31 (m, 7H), 6.81 (s, 1H), 5.91 (s, 1H), 1.38 (s, 9H). ^13^C NMR (101 MHz, CDCl_3_) δ 166.15, 155.49, 149.23, 133.93, 133.91, 130.96, 129.33, 128.73, 128.47, 127.95, 125.33, 124.37, 123.78, 122.84, 122.02, 121.32, 118.77, 118.04, 117.80, 61.13, 52.26, 28.55. HRMS (ESI) *m*/*z* calcd. for C_26_H_24_ClN_4_O_2_^+^ (M + H)^+^ 459.1582, found 459.1557.

*N*-(*tert*-butyl)-2-(9-chloro-6-oxoindazolo[2,3-*a*]quinoxalin-5(6*H*)-yl)-2-phenylacetamide (**6r**), ^1^H NMR (400 MHz, CDCl_3_) δ 8.55 (dd, *J* = 6.2, 3.5 Hz, 1H), 8.17 (d, *J* = 8.8 Hz, 1H), 7.90–7.84 (m, 1H), 7.56 (dd, *J* = 6.2, 3.4 Hz, 1H), 7.45–7.29 (m, 7H), 7.25 (dd, *J* = 5.1, 1.8 Hz, 1H), 6.92 (s, 1H), 6.19 (s, 1H), 1.34 (s, 9H). ^13^C NMR (101 MHz, CDCl_3_) δ 166.59, 155.42, 149.22, 134.17, 134.13, 129.94, 129.06, 128.42, 128.32, 127.88, 126.28, 124.92, 124.39, 123.33, 122.39, 120.13, 118.40, 117.66, 116.85, 60.78, 52.17, 28.58. HRMS (ESI) *m*/*z* calcd. for C_26_H_24_ClN_4_O_2_^+^ (M + H)^+^ 459.1582, found 459.1583.

*N*-(*tert*-butyl)-2-(9-chloro-2-methyl-6-oxoindazolo[2,3-*a*]quinoxalin-5(6*H*)-yl)-2-phenylacetamide (**6s**), ^1^H NMR (400 MHz, CDCl_3_) δ 8.49 (d, *J* = 8.4 Hz, 1H), 8.28 (d, *J* = 8.8 Hz, 1H), 7.93 (d, *J* = 1.1 Hz, 1H), 7.45–7.31 (m, 7H), 7.21 (d, *J* = 8.4 Hz, 1H), 6.80 (s, 1H), 5.94 (s, 1H), 2.37 (s, 3H), 1.35 (s, 9H). ^13^C NMR (101 MHz, CDCl_3_) δ 166.54, 155.44, 149.21, 139.05, 134.13, 134.04, 130.03, 129.08, 128.46, 127.95, 126.17, 125.50, 123.14, 122.92, 122.52, 120.21, 118.00, 117.57, 116.77, 61.16, 52.11, 28.53, 21.81. HRMS (ESI) *m*/*z* calcd. for C_27_H_26_ClN_4_O_2_^+^ (M + H)^+^ 473.1739, found 473.1733.

*N*-benzyl-2-(3-bromophenyl)-2-(9-chloro-6-oxoindazolo[2,3-*a*]quinoxalin-5(6*H*)-yl)acetamide (**6t**), ^1^H NMR (400 MHz, CDCl_3_) δ 8.56–8.48 (m, 1H), 7.90–7.82 (m, 2H), 7.57 (s, 1H), 7.51–7.42 (m, 2H), 7.41–7.35 (m, 2H), 7.24 (s, 1H), 7.20–7.09 (m, 7H), 6.99 (s, 2H), 4.54–4.41 (m, 2H). ^13^C NMR (101 MHz, CDCl_3_) δ 166.89, 155.38, 149.12, 137.42, 135.73, 134.29, 131.59, 130.97, 130.38, 129.17, 128.66, 128.59, 127.73, 127.55, 126.57, 126.38, 125.01, 124.81, 123.05, 122.83, 121.83, 119.97, 118.22, 117.88, 116.96, 58.45, 44.13. HRMS (ESI) *m*/*z* calcd. for C_29_H_21_BrClN_4_O_2_^+^ (M + H)^+^ 571.0531, found 571.0534.

*N*-(*tert*-butyl)-2-(8-methyl-6-oxoindazolo[2,3-*a*]quinoxalin-5(6*H*)-yl)-2-phenylacetamide (**6u**), ^1^H NMR (400 MHz, CDCl_3_) δ 8.62 (dd, *J* = 7.9, 1.6 Hz, 1H), 8.14 (s, 1H), 7.87 (d, *J* = 8.8 Hz, 1H), 7.55 (dd, *J* = 8.0, 1.2 Hz, 1H), 7.37 (ddt, *J* = 18.2, 10.2, 7.2 Hz, 8H), 6.91 (s, 1H), 6.02 (s, 1H), 2.54 (s, 3H), 1.35 (s, 9H). ^13^C NMR (101 MHz, CDCl_3_) δ 166.79, 155.85, 148.07, 135.20, 134.22, 131.12, 129.75, 128.93, 128.22, 127.87, 127.83, 125.32, 124.22, 122.33, 122.25, 119.64, 118.29, 117.48, 117.43, 60.51, 52.08, 28.58, 22.00. HRMS (ESI) *m*/*z* calcd. for C_27_H_27_N_4_O_2_^+^ (M + H)^+^ 439.2129, found 439.2119.

*N*-benzyl-2-(6-oxoindazolo[2,3-*a*]quinoxalin-5(6*H*)-yl)acetamide (**6v**), ^1^H NMR (400 MHz, DMSO-*d*_6_) δ 8.76 (s, 1H), 8.58 (d, *J* = 7.3 Hz, 1H), 8.27 (d, *J* = 7.5 Hz, 1H), 8.00 (d, *J* = 7.9 Hz, 1H), 7.71–7.40 (m, 5H), 7.38–7.14 (m, 5H), 5.10 (s, 2H), 4.31 (s, 2H). ^13^C NMR (101 MHz, DMSO-*d*_6_) δ 167.08, 154.93, 148.74, 139.52, 131.13, 129.47, 128.73, 127.56, 127.30, 125.24, 124.66, 124.45, 123.68, 121.21, 121.18, 118.05, 117.43, 116.72, 44.74, 42.62. HRMS (ESI) *m*/*z* calcd. for C_23_H_19_N_4_O_2_^+^ (M + H)^+^ 383.1503, found 383.1487.

## 4. Conclusions

In summary, we developed a facile and efficient methodology for the construction of an indazole-fused polyheterocycle using a unique Ugi/Ullmann cascade reaction strategy. With this protocol, complex functionalized indazolo-quinoxaline derivatives were obtained in good to excellent yields in one pot, with excellent functional group tolerance. Anticancer activity evaluation revealed that the synthesized new compounds show potential anticancer activities that may have a wide range of applications in organic synthesis and medicinal chemistry.

## Figures and Tables

**Table 1:** Optimization of the Ullmann reaction conditions from **5a** ^a^.

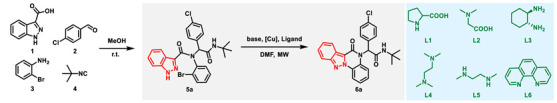						
Entry	Base (2.0 Eqiv.)	Cat. (10 mol%)	Ligand (20 mol%)	Temp. (°C)	Time (Min)	Yield of 6a (%) ^b^
1	K_2_CO_3_	/	/	MW 120	10 min	Trace
2	K_2_CO_3_	/	/	MW 150	10 min	25
3	K_2_CO_3_	/	/	MW 150	30 min	55
4	KOtBu	/	/	MW 150	30 min	31
5	Cs_2_CO_3_	/	/	MW 150	30 min	43
6	TEA	/	/	MW 150	30 min	Trace
7	DBU	/	/	MW 150	30 min	15
8	DMAP	/	/	MW 150	30 min	17
9	K_2_CO_3_	CuI	L1	MW 150	30 min	57
10	K_2_CO_3_	CuI	L2	MW 150	30 min	67
11	K_2_CO_3_	CuI	L3	MW 150	30 min	79
12	K_2_CO_3_	CuI	L4	MW 150	30 min	95
13	K_2_CO_3_	CuI	L5	MW 150	30 min	83
14	K_2_CO_3_	CuI	L6	MW 150	30 min	73
15	K_2_CO_3_	CuBr	L4	MW 150	30 min	65
16	K_2_CO_3_	Cu_2_O	L4	MW 150	30 min	53
17	K_2_CO_3_	Cu(OTf)_2_	L4	MW 150	30 min	49

^a^ Reaction conditions: **5a** (0.1 mmol) and base (0.2 mmol) in DMF (1.0 mL) under air. ^b^ Isolated yields. MW = microwave irradiation.

## Data Availability

All the required data are reported in the manuscript and Supplementary Materials.

## Supplementary Materials

The following supporting information can be downloaded at: https://www.mdpi.com/article/10.3390/molecules29020464/s1, X-ray of **6k**; copies of ^1^H, ^13^C NMR spectra of compounds **6**.
