# Supplementary material for: A Facile Ugi/Ullmann Cascade Reaction to Access Fused Indazolo-Quinoxaline Derivatives with Potent Anticancer Activity
Source: Molecules. 2024 Jan 17;29(2):464. doi: 10.3390/molecules29020464 (PMC10820152; doi:10.3390/molecules29020464)

# checkCIF/PLATON report

Structure factors have been supplied for datablock(s) 1

THIS REPORT IS FOR GUIDANCE ONLY. IF USED AS PART OF A REVIEW PROCEDURE FOR PUBLICATION, IT SHOULD NOT REPLACE THE EXPERTISE OF AN EXPERIENCED CRYSTALLOGRAPHIC REFEREE.

No syntax errors found. CIF dictionary Interpreting this report

# Datablock: 1

---

| | | | |
|---|---|---|---|
| Bond precision: | C-C = 0.0057 A | Wavelength=0.71073 | |
| Cell: | a=13.644(12) | b=25.64(2) | c=29.54(3) |
| | alpha=90 | beta=90 | gamma=90 |
| Temperature: | 296 K | | |

| | Calculated | Reported |
|---|---|---|
| Volume | 10334(16) | 10334(16) |
| Space group | P b c n | P b c n |
| Hall group | -P 2n 2ab | -P 2n 2ab |
| Moiety formula | 4(C28 H25 Cl N4 O2), H2 O | ? |
| Sum formula | C112 H102 Cl4 N16 O9 | C56 H51 Cl2 N8 O4.50 |
| Mr | 1957.90 | 978.95 |
| Dx,g cm-3 | 1.258 | 1.258 |
| Z | 4 | 8 |
| Mu (mm-1) | 0.181 | 0.181 |
| F000 | 4104.0 | 4104.0 |
| F000’ | 4107.89 | |
| h,k,lmax | 16,30,35 | 16,30,35 |
| Nref | 9110 | 9086 |
| Tmin,Tmax | 0.957,0.971 | |
| Tmin’ | 0.954 | |

Correction method= Not given

Data completeness= 0.997 Theta(max)= 24.999

R(reflections)= 0.0572( 3732) wR2(reflections)= 0.1455( 9086)

S = 0.850 Npar= 640

The following ALERTS were generated. Each ALERT has the format
**test-name_ALERT_alert-type_alert-level.**
Click on the hyperlinks for more details of the test.

## Alert level C

```
RINTA01_ALERT_3_C  The value of Rint is greater than 0.12
            Rint given   0.153
PLAT026_ALERT_3_C Ratio Observed / Unique Reflections (too) Low ..       41% Check
PLAT148_ALERT_3_C s.u. on the      c    - Axis is (Too) Large ....     0.030 Ang.
PLAT220_ALERT_2_C NonSolvent   Resd 1  C   Ueq(max)/Ueq(min) Range       3.6 Ratio
PLAT234_ALERT_4_C Large Hirshfeld Difference C3       --C4       .      0.17 Ang.
PLAT241_ALERT_2_C High   'MainMol' Ueq as Compared to Neighbors of        C4 Check
PLAT242_ALERT_2_C Low    'MainMol' Ueq as Compared to Neighbors of        C6 Check
PLAT242_ALERT_2_C Low    'MainMol' Ueq as Compared to Neighbors of       C12 Check
PLAT242_ALERT_2_C Low    'MainMol' Ueq as Compared to Neighbors of       C40 Check
PLAT260_ALERT_2_C Large Average Ueq of Residue Including        O5     0.269 Check
PLAT340_ALERT_3_C Low Bond Precision on  C-C Bonds ...............   0.00566 Ang.
PLAT601_ALERT_2_C Unit Cell Contains Solvent Accessible VOIDS of .        42 Ang**3
PLAT906_ALERT_3_C Large K Value in the Analysis of Variance ......    31.569 Check
PLAT906_ALERT_3_C Large K Value in the Analysis of Variance ......     5.275 Check
PLAT910_ALERT_3_C Missing # of FCF Reflection(s) Below Theta(Min).         9 Note
PLAT911_ALERT_3_C Missing FCF Refl Between Thmin & STh/L=    0.595        17 Report
```

## Alert level G

```
PLAT003_ALERT_2_G Number of Uiso or Uij Restrained non-H Atoms ...         2 Report
PLAT007_ALERT_5_G Number of Unrefined Donor-H Atoms ..............         4 Report
PLAT020_ALERT_3_G The Value of Rint is Greater Than 0.12 .........     0.153 Report
PLAT045_ALERT_1_G Calculated and Reported Z Differ by a Factor ...     0.500 Check
PLAT177_ALERT_4_G The CIF-Embedded .res File Contains DELU Records         1 Report
PLAT192_ALERT_3_G A Non-default DELU Restraint Value for First Par    0.0010 Report
PLAT192_ALERT_3_G A Non-default DELU Restraint Value for SecondPar    0.0020 Report
PLAT300_ALERT_4_G Atom Site Occupancy of H5C        Constrained at       0.5 Check
PLAT300_ALERT_4_G Atom Site Occupancy of H5D        Constrained at       0.5 Check
PLAT793_ALERT_4_G Model has Chirality at C8          (Centro SPGR)         R Verify
PLAT793_ALERT_4_G Model has Chirality at C36         (Centro SPGR)         S Verify
PLAT860_ALERT_3_G Number of Least-Squares Restraints .............         1 Note
PLAT883_ALERT_1_G No Info/Value for _atom_sites_solution_primary .    Please Do !
PLAT913_ALERT_3_G Missing # of Very Strong Reflections in FCF ....         3 Note
PLAT933_ALERT_2_G Number of HKL-OMIT Records in Embedded .res File        22 Note
PLAT961_ALERT_5_G Dataset Contains no Negative Intensities .......    Please Check
PLAT967_ALERT_5_G Note: Two-Theta Cutoff Value in Embedded .res ..      50.0 Degree
PLAT978_ALERT_2_G Number C-C Bonds with Positive Residual Density.         4 Info
```

 0 **ALERT level A** = Most likely a serious problem - resolve or explain
 0 **ALERT level B** = A potentially serious problem, consider carefully
16 **ALERT level C** = Check. Ensure it is not caused by an omission or oversight
18 **ALERT level G** = General information/check it is not something unexpected

 2 ALERT type 1 CIF construction/syntax error, inconsistent or missing data
10 ALERT type 2 Indicator that the structure model may be wrong or deficient
13 ALERT type 3 Indicator that the structure quality may be low
 6 ALERT type 4 Improvement, methodology, query or suggestion
 3 ALERT type 5 Informative message, check

It is advisable to attempt to resolve as many as possible of the alerts in all categories. Often the minor alerts point to easily fixed oversights, errors and omissions in your CIF or refinement strategy, so attention to these fine details can be worthwhile. In order to resolve some of the more serious problems it may be necessary to carry out additional measurements or structure refinements. However, the purpose of your study may justify the reported deviations and the more serious of these should normally be commented upon in the discussion or experimental section of a paper or in the "special_details" fields of the CIF. checkCIF was carefully designed to identify outliers and unusual parameters, but every test has its limitations and alerts that are not important in a particular case may appear. Conversely, the absence of alerts does not guarantee there are no aspects of the results needing attention. It is up to the individual to critically assess their own results and, if necessary, seek expert advice.

**Publication of your CIF in IUCr journals**

A basic structural check has been run on your CIF. These basic checks will be run on all CIFs submitted for publication in IUCr journals (*Acta Crystallographica*, *Journal of Applied Crystallography*, *Journal of Synchrotron Radiation*); however, if you intend to submit to *Acta Crystallographica Section C* or *E* or *IUCrData*, you should make sure that full publication checks are run on the final version of your CIF prior to submission.

**Publication of your CIF in other journals**

Please refer to the *Notes for Authors* of the relevant journal for any special instructions relating to CIF submission.

**PLATON version of 28/11/2022; check.def file version of 28/11/2022**

**Datablock 1** - ellipsoid plot

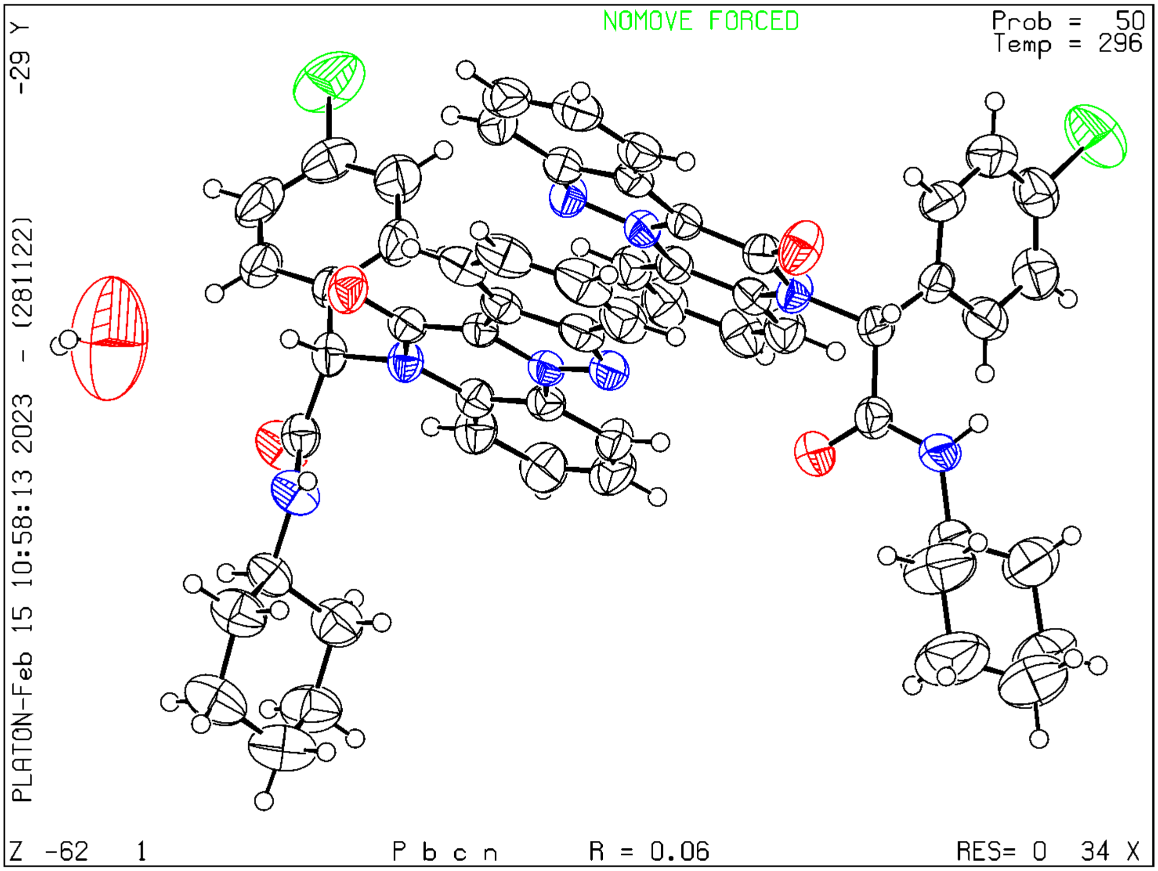

Supplement: Supplementary file 1 [file molecules-29-00464-s001.zip › molecules-2786104-supplementary/checkcif.pdf]
