# Supplementary material for: A Facile Ugi/Ullmann Cascade Reaction to Access Fused Indazolo-Quinoxaline Derivatives with Potent Anticancer Activity
Source: Molecules. 2024 Jan 17;29(2):464. doi: 10.3390/molecules29020464 (PMC10820152; doi:10.3390/molecules29020464)

Supporting Information

A Facile Ugi/Ullmann Cascade Reaction to Access Fused Indazolo-Quinoxaline Derivatives with Potent Anticancer Activity

Yong Li [1,2], Liu-Jun He[1], Hong-Xia Qin[1]*, Yao Liu[1], Bin-Xin Yang[1], Zhi-Gang Xu[1], Dong-Lin Yang[1]*

[1] College of Pharmacy, National & Local Joint Engineering Research Center of Targeted and Innovative Therapeutics, Chongqing Key Laboratory of Kinase Modulators as Innovative Medicine, Chongqing University of Arts and Sciences, Chongqing 402160, China

[2] School of Chemistry and Chemical Engineering, Southwest University, Chongqing 400715, China

Correspondence: 17320489614@163.com (H.-X.Q.)
dlyang@cqwu.edu.cn (D.-L.Y.)

Table of Contents

1. X-Ray crystal data for compound **6k**...S2

2. ^{1}H, and ^{13}C NMR spectra for compounds **6**...S3

1. X-Ray crystal data for compounds **6k**

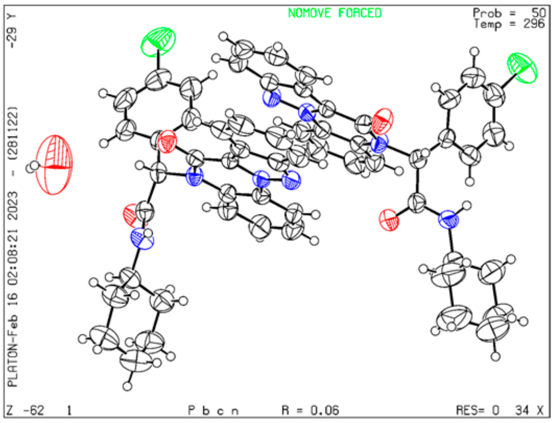

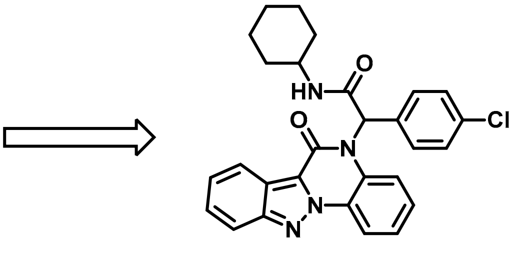

6k (CCDC 2242210)

2. ^{1}H and ^{13}C NMR spectra for compounds **6**

^{1}H NMR spectrum of **6a (**400 MHz, $CDCl_3$**)**

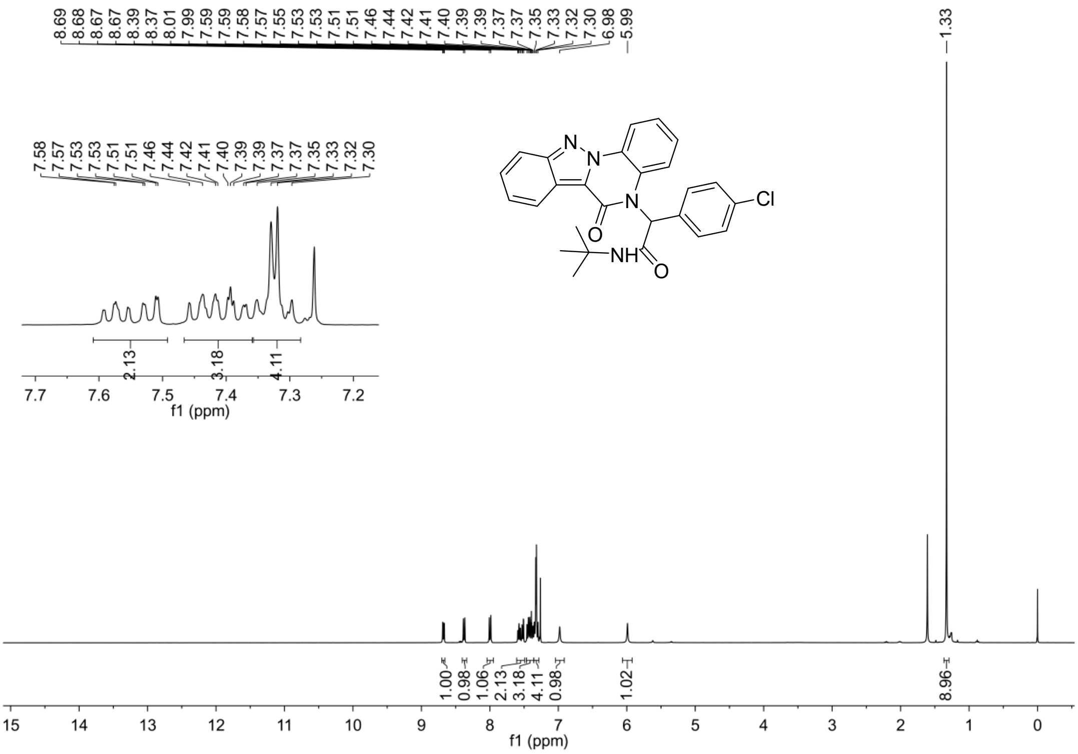

^{13}C NMR spectrum of **6a (**101 MHz, $CDCl_3$**)**

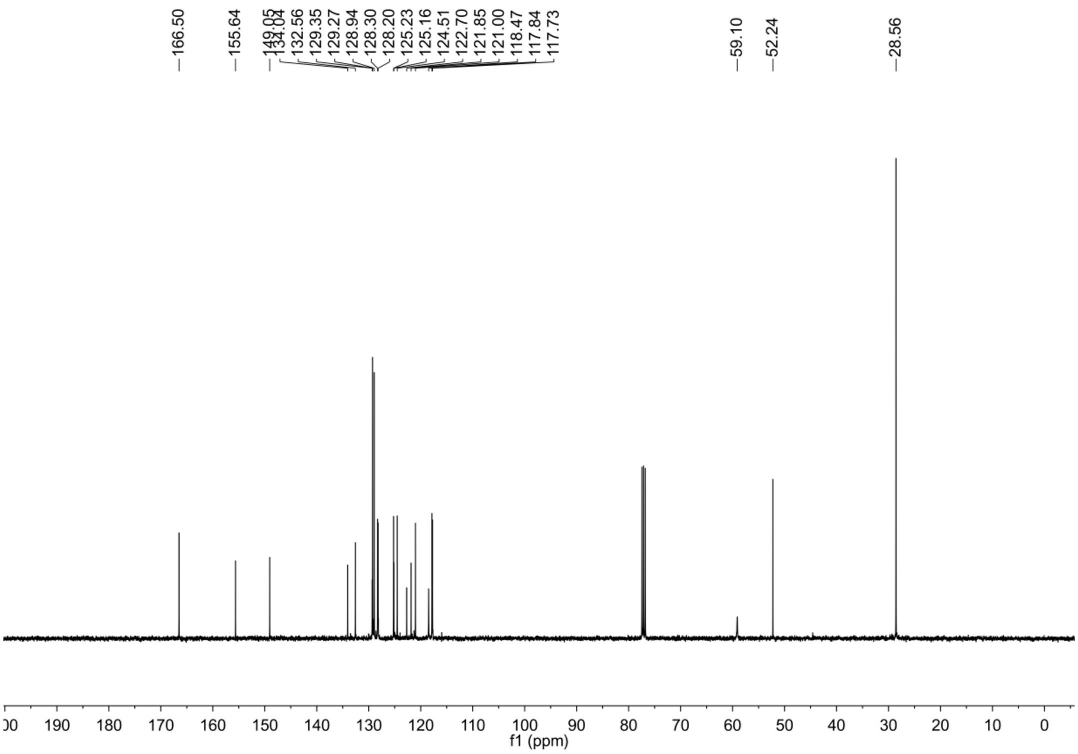

^{1}H NMR spectrum of **6b (**400 MHz, $CDCl_3$**)**

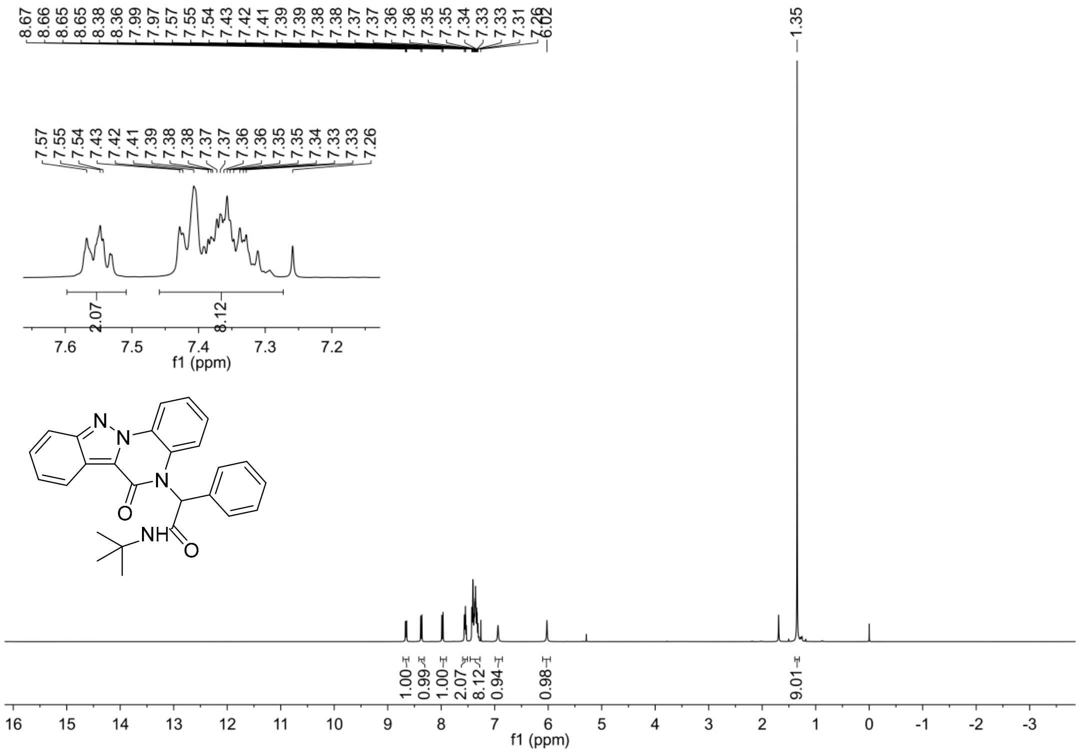

^{13}C NMR spectrum of **6b (**101 MHz, $CDCl_3$**)**

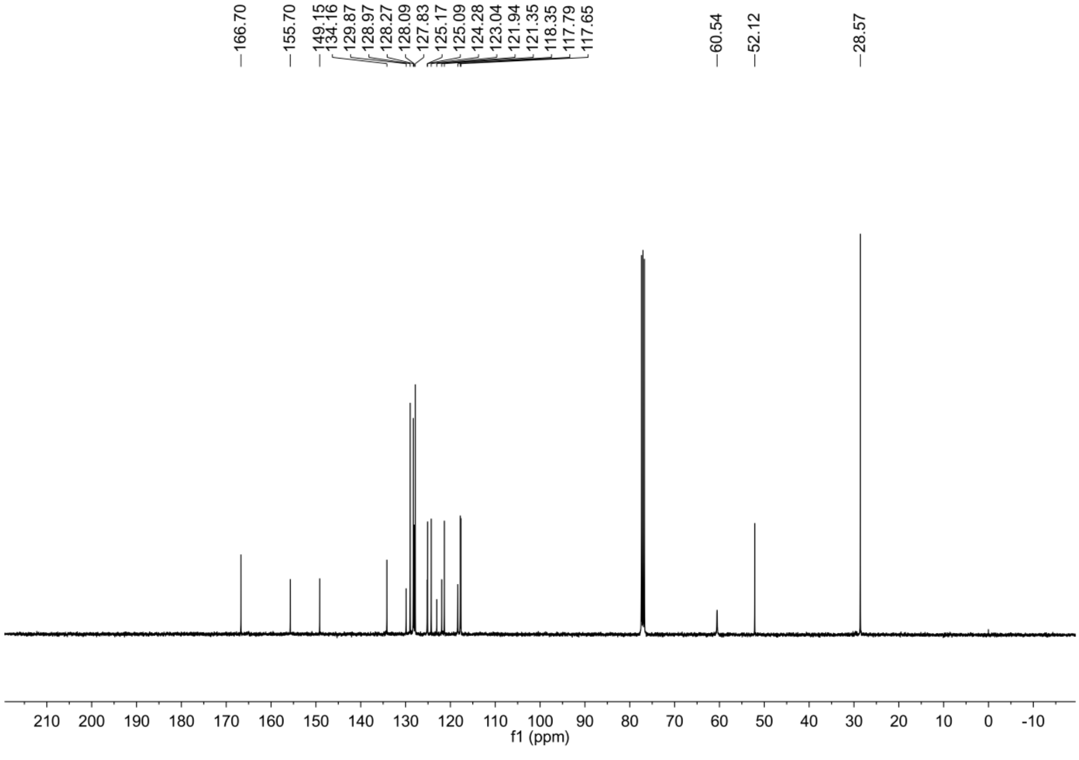

^{1}H NMR spectrum of **6c (**400 MHz, CDCl$_3$**)**

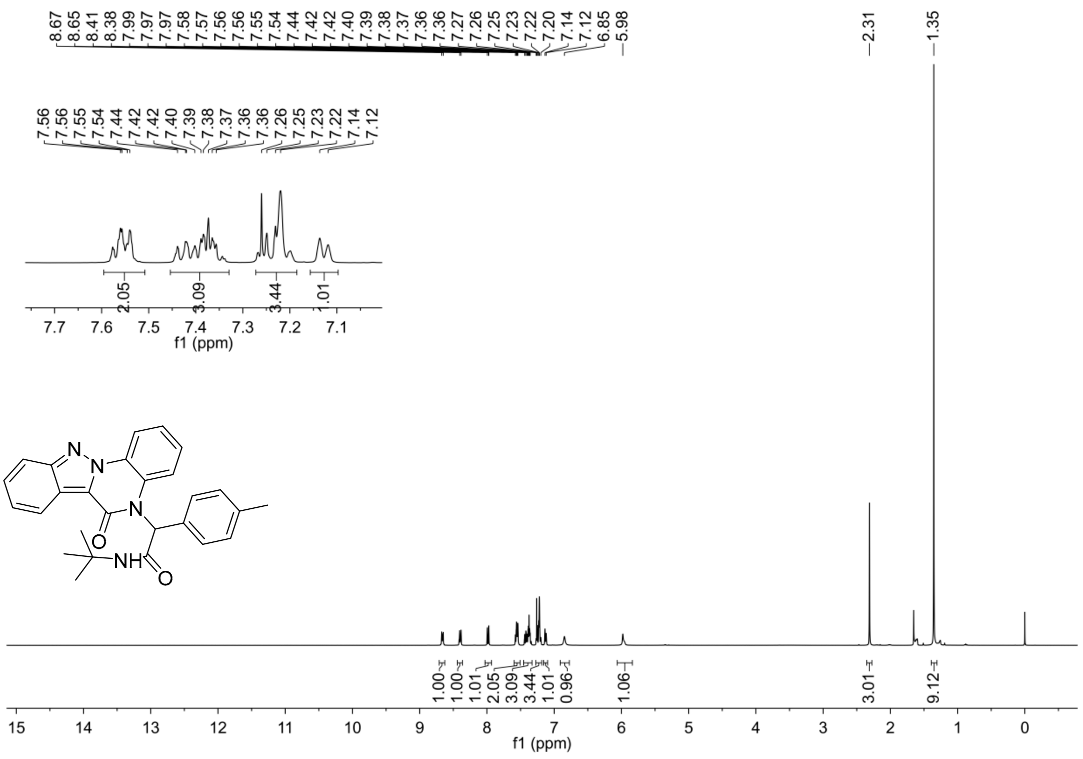

^{13}C NMR spectrum of **6c (**101 MHz, CDCl$_3$**)**

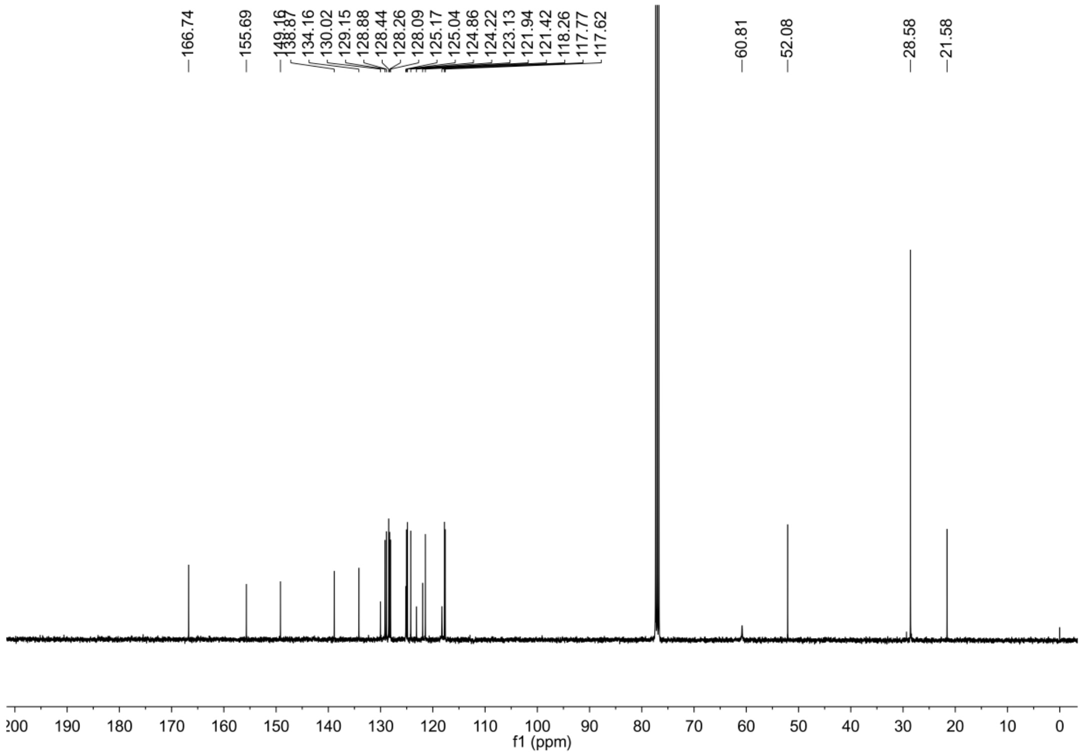

^{1}H NMR spectrum of **6d (**400 MHz, $CDCl_3$**)**

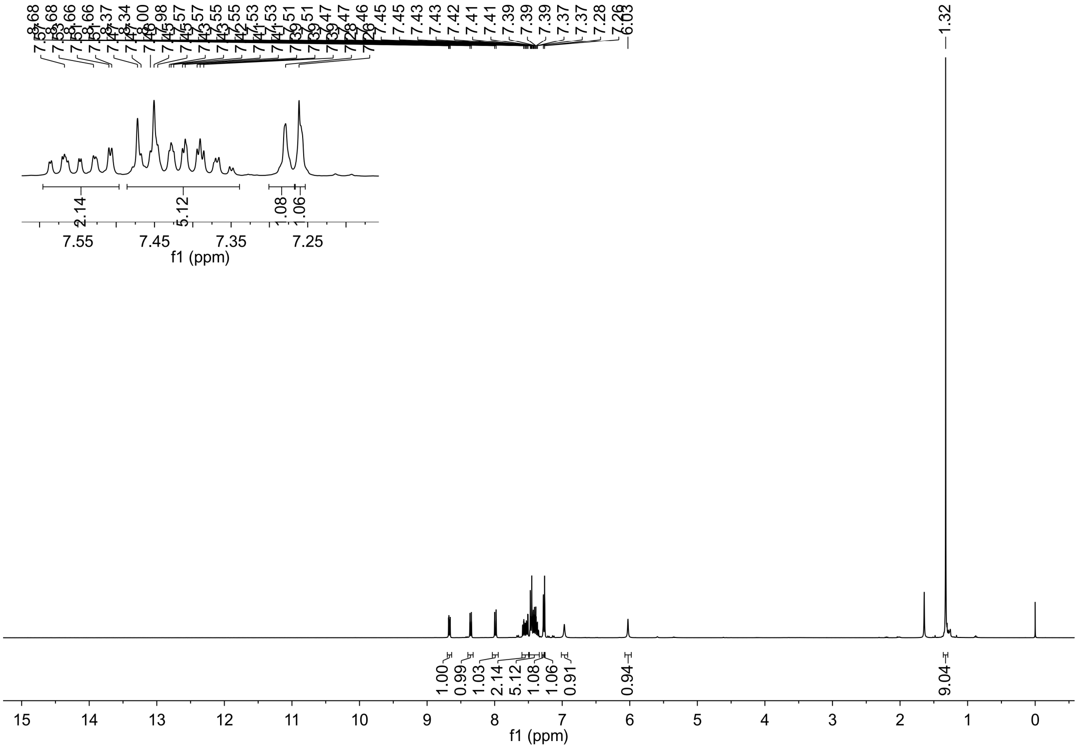

^{13}C NMR spectrum of **6d (**101 MHz, $CDCl_3$**)**

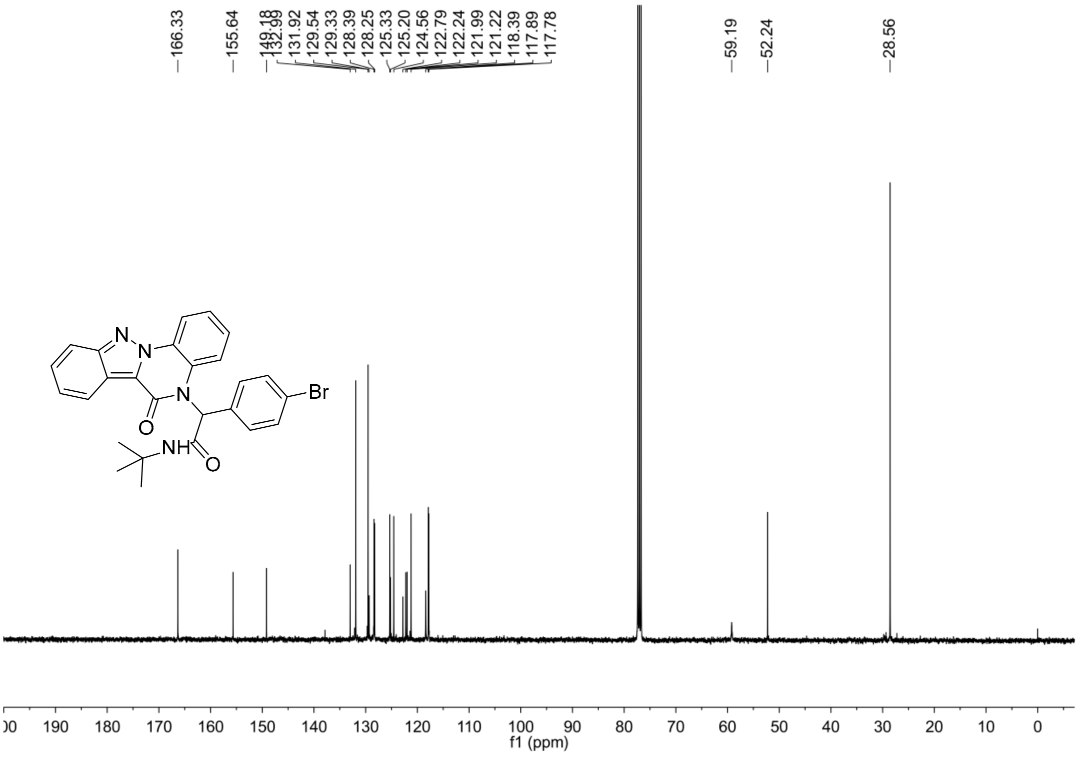

^{1}H NMR spectrum of **6e (**400 MHz, $CDCl_3$**)**

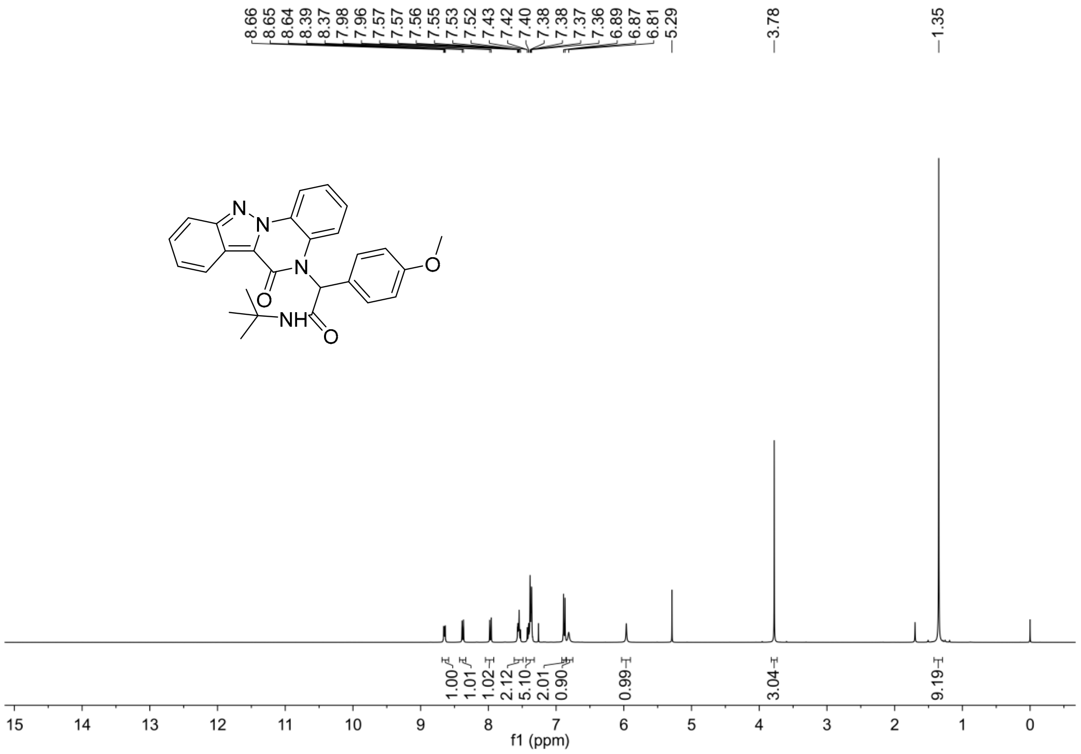

^{13}C NMR spectrum of **6e (**101 MHz, $CDCl_3$**)**

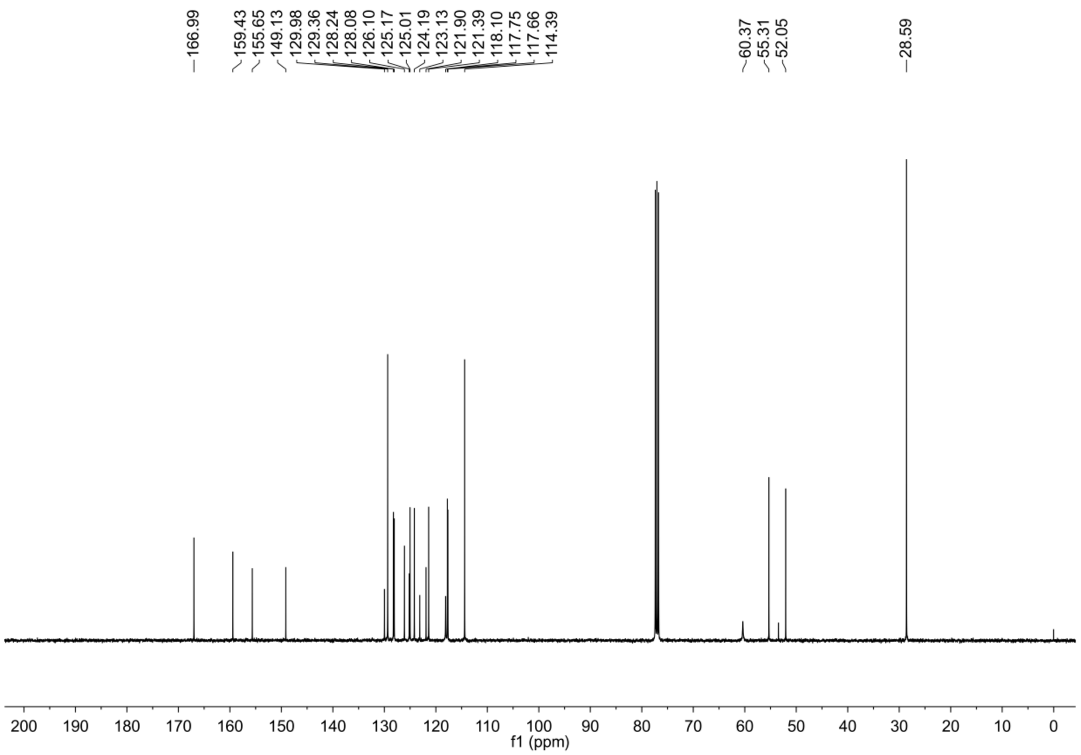

^{1}H NMR spectrum of **6f (**101 MHz, CDCl$_3$**)**

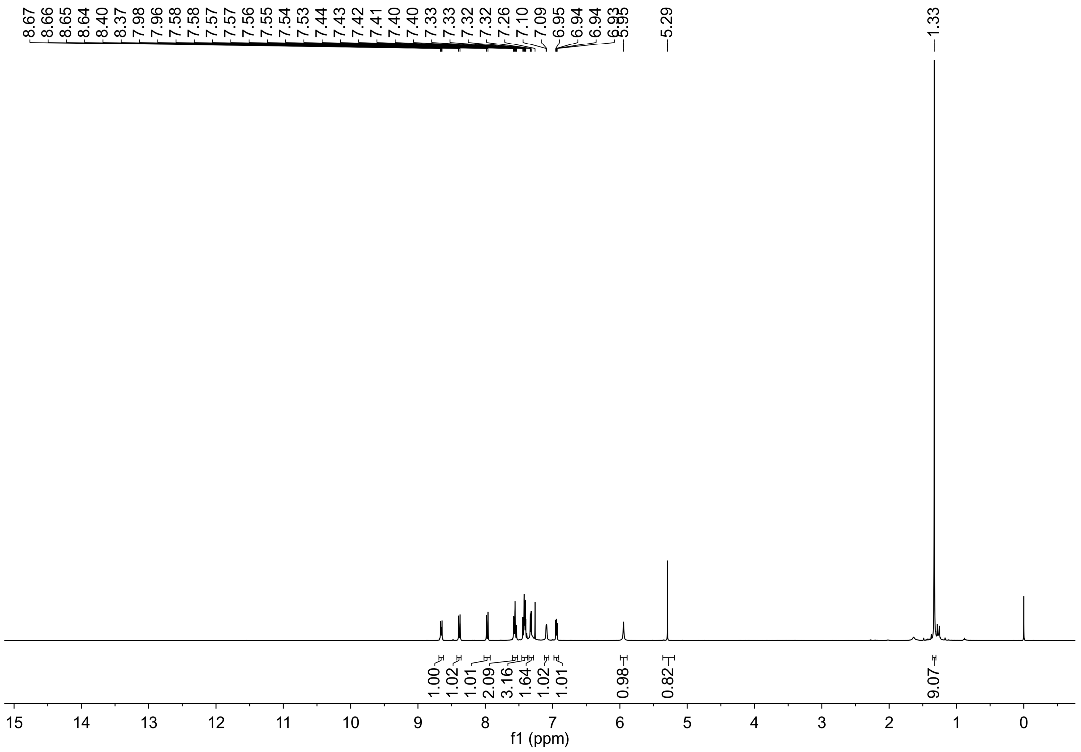

^{13}C NMR spectrum of **6f (**101 MHz, CDCl$_3$**)**

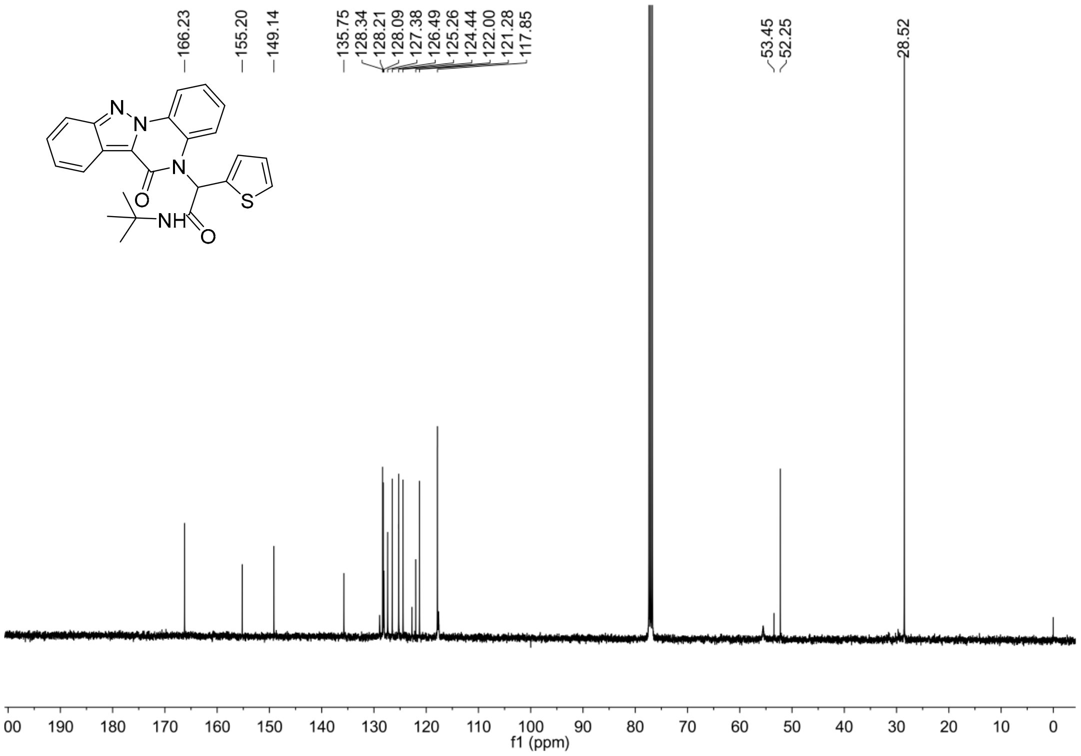

^{1}H NMR spectrum of **6g** (400 MHz, $CDCl_3$)

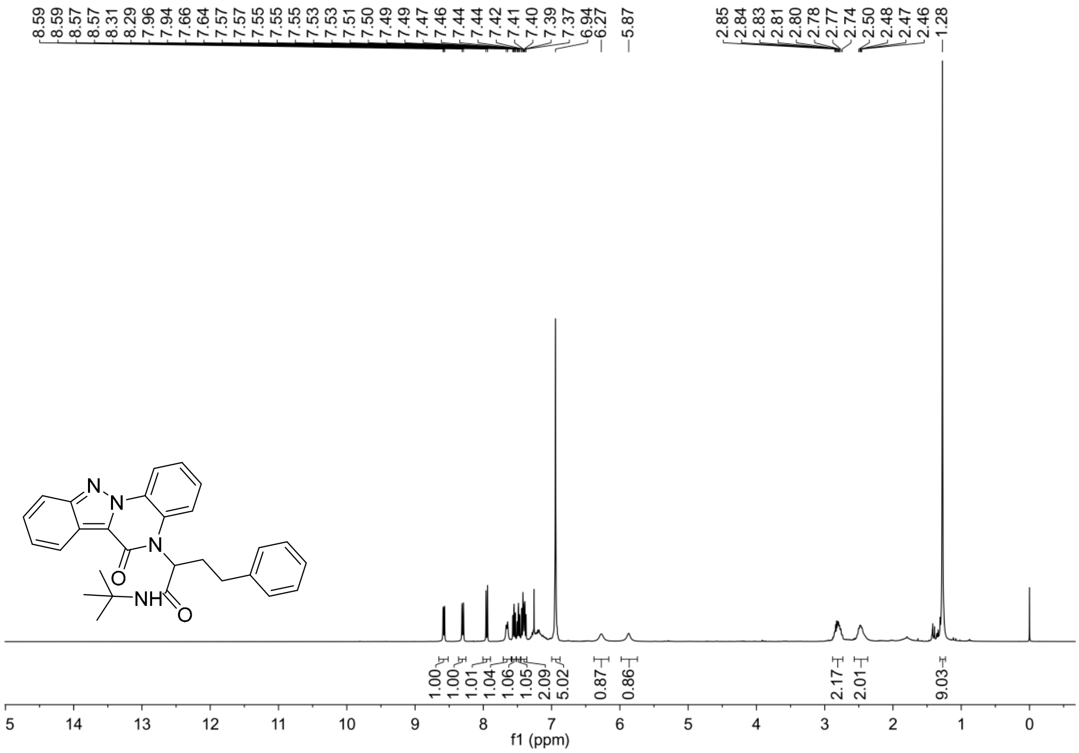

^{13}C NMR spectrum of **6g** (101 MHz, $CDCl_3$)

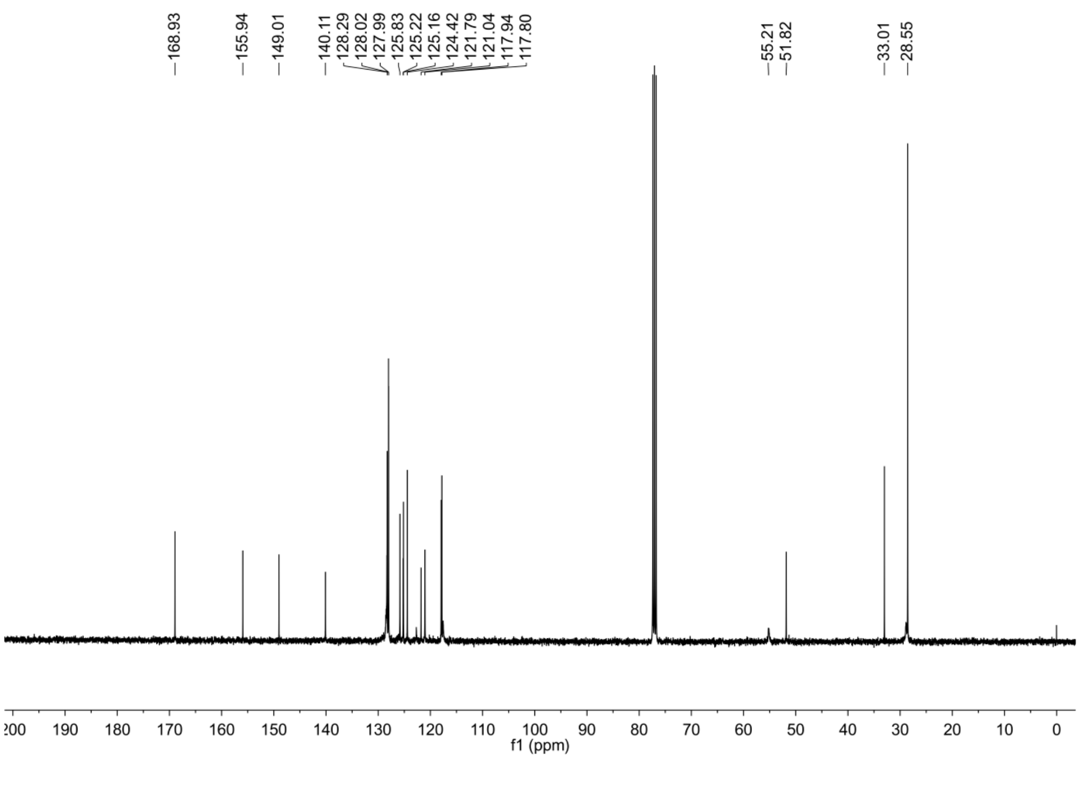

^1H NMR spectrum of **6h (**400 MHz, $CDCl_3$**)**

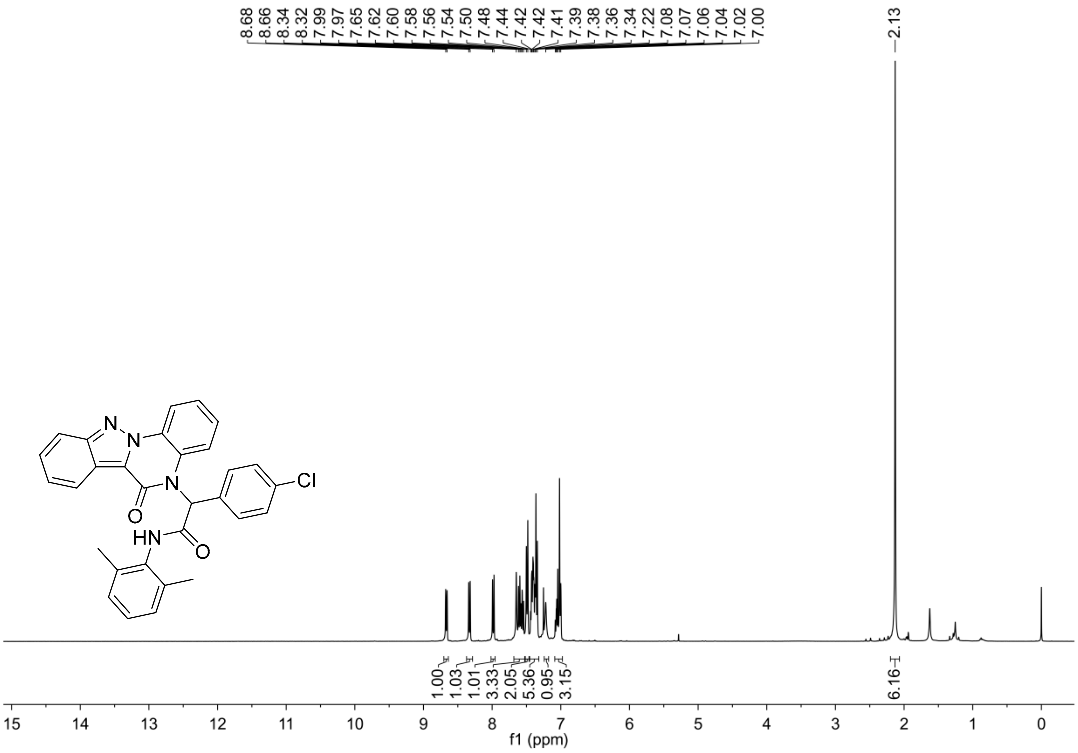

^{13}C NMR spectrum of **6h (**101 MHz, $CDCl_3$**)**

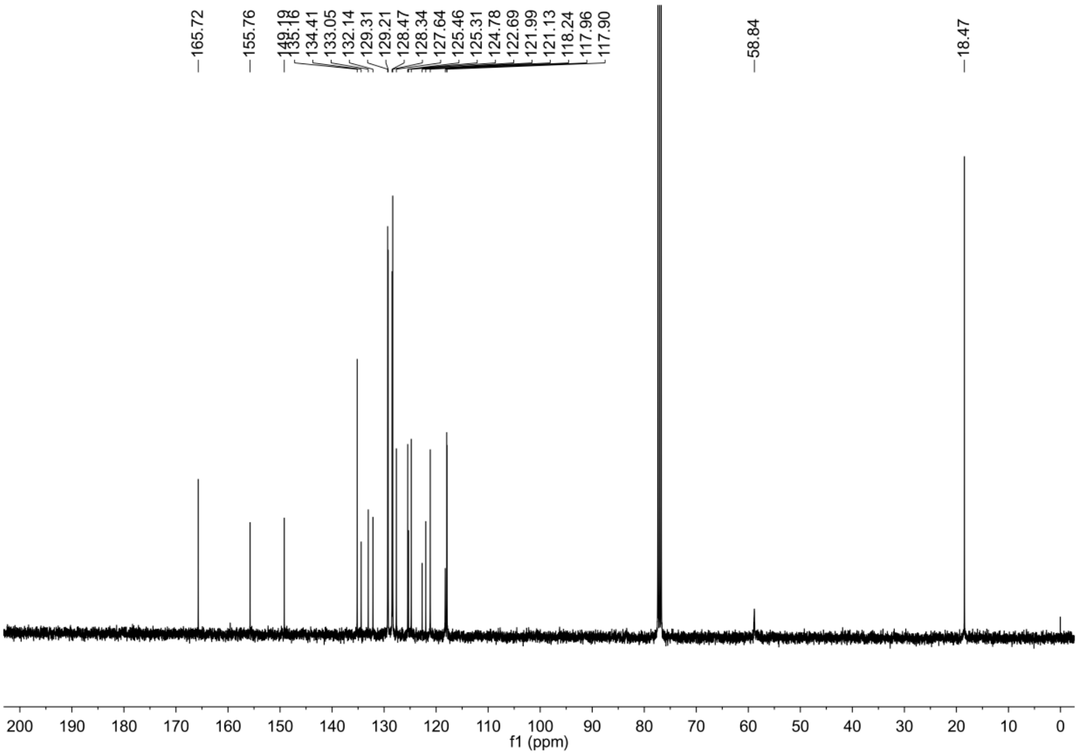

^{1}H NMR spectrum of **6i (**400 MHz, $CDCl_3$**)**

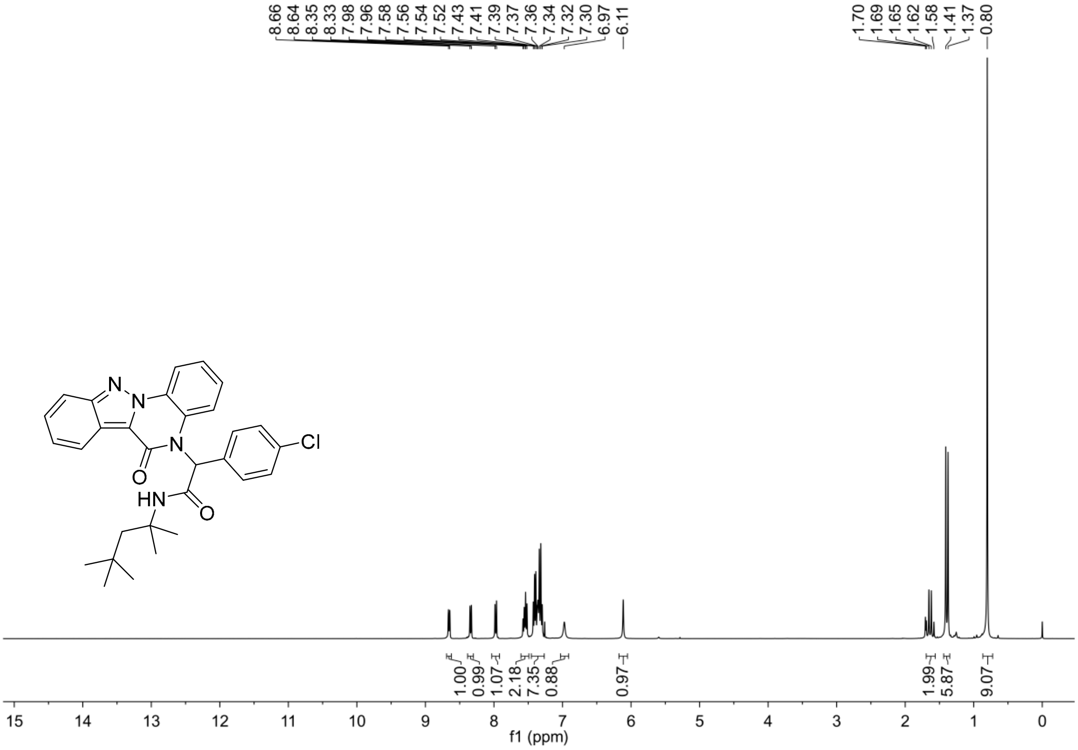

^{13}C NMR spectrum of **6i (**101 MHz, $CDCl_3$**)**

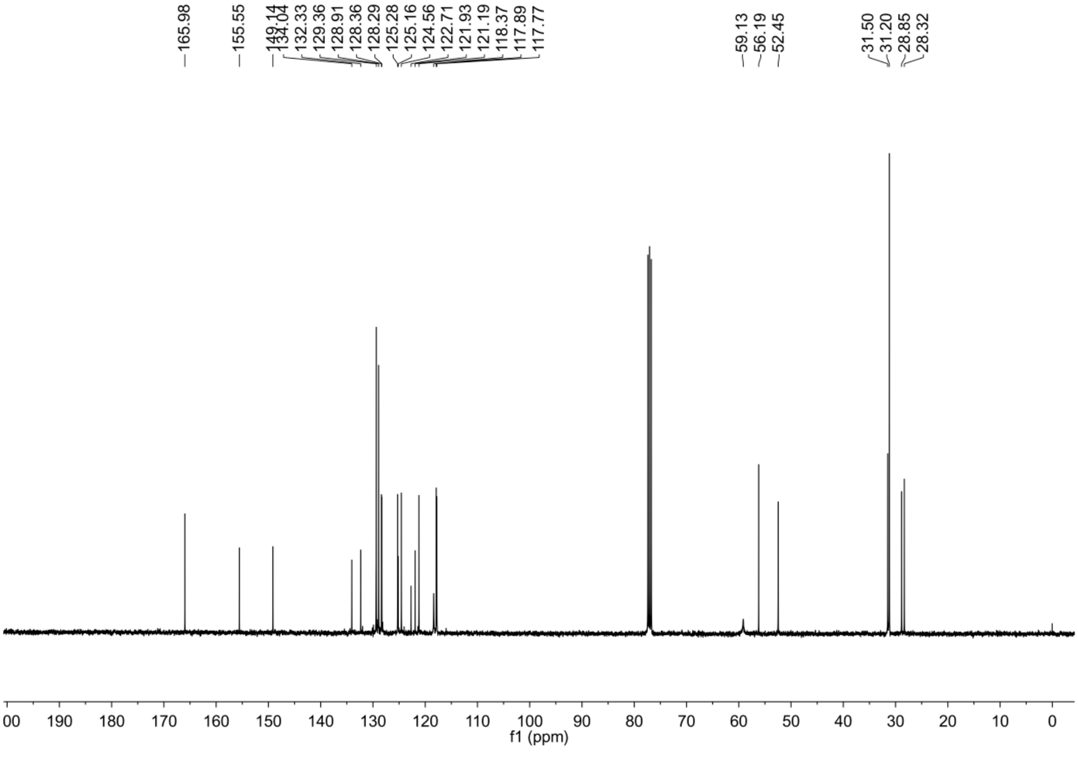

^{1}H NMR spectrum of **6j** (400 MHz, $CDCl_3$)

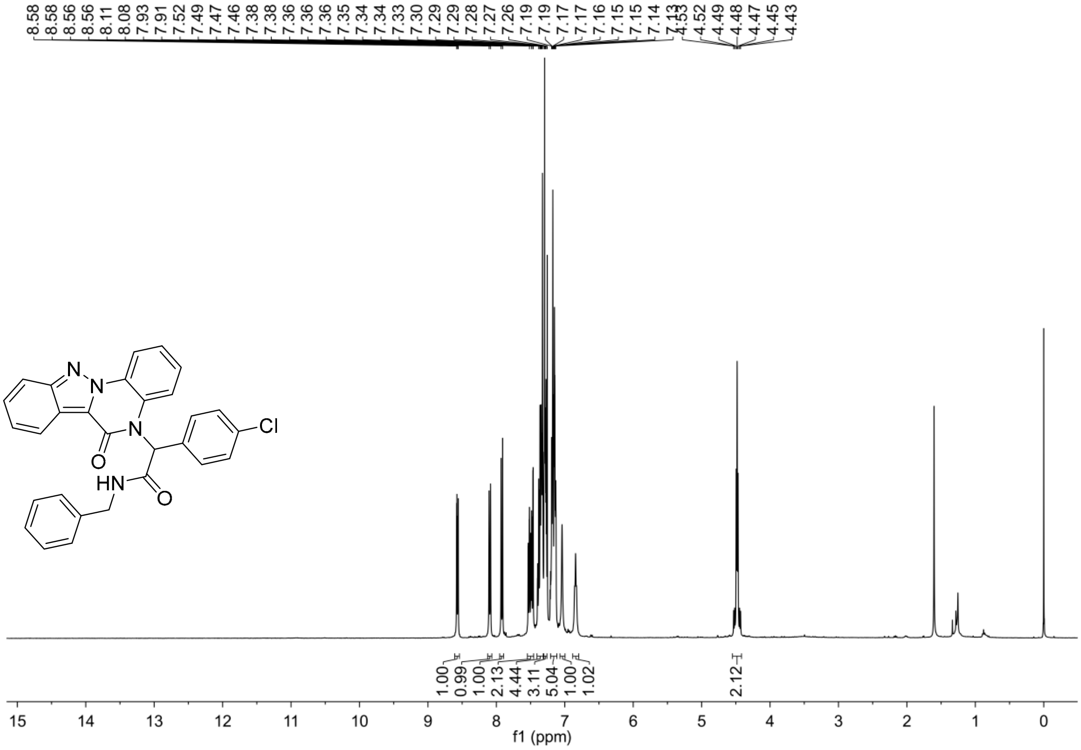

^{13}C NMR spectrum of **6j** (101 MHz, $CDCl_3$)

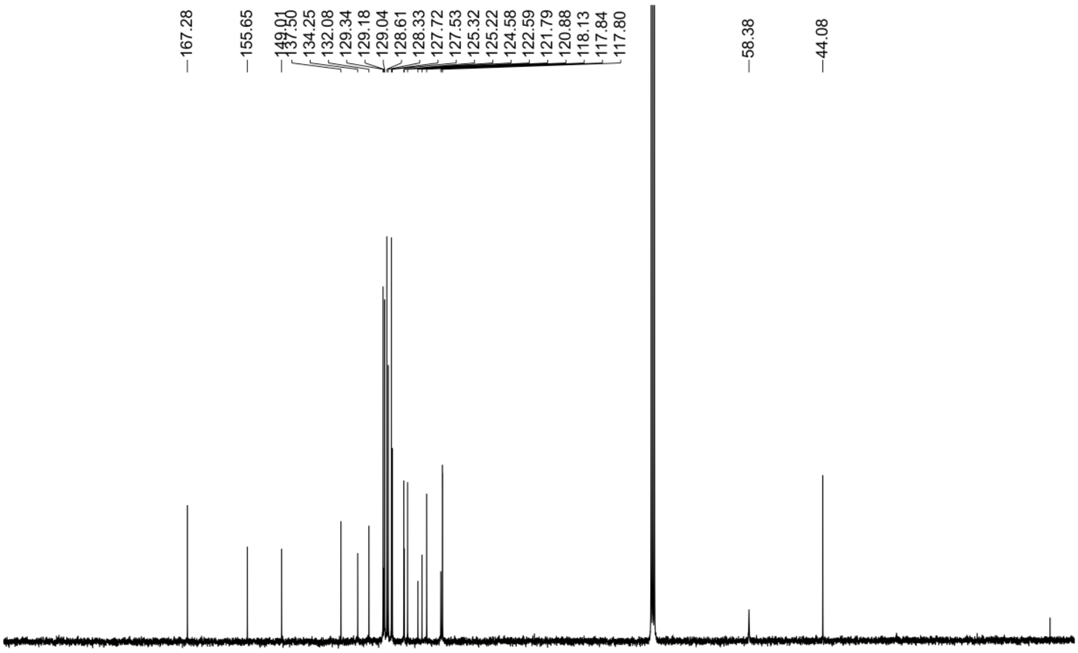

^{1}H NMR spectrum of **6k (**400 MHz, $CDCl_3$**)**

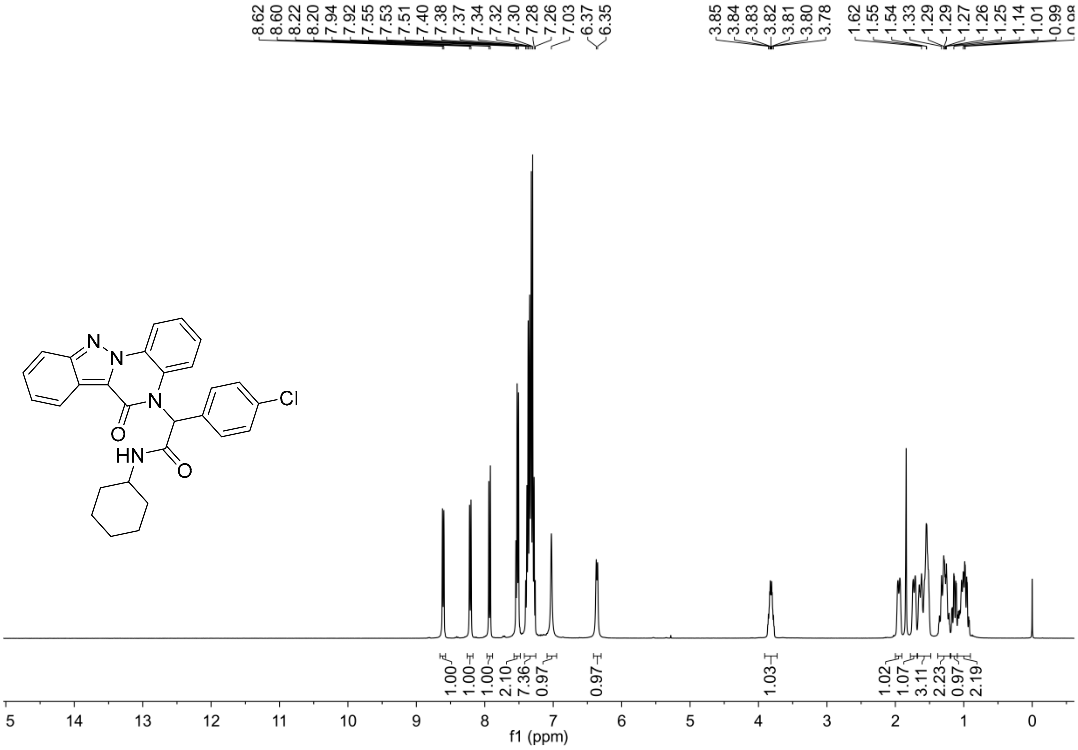

^{13}C NMR spectrum of **6k (**101 MHz, $CDCl_3$**)**

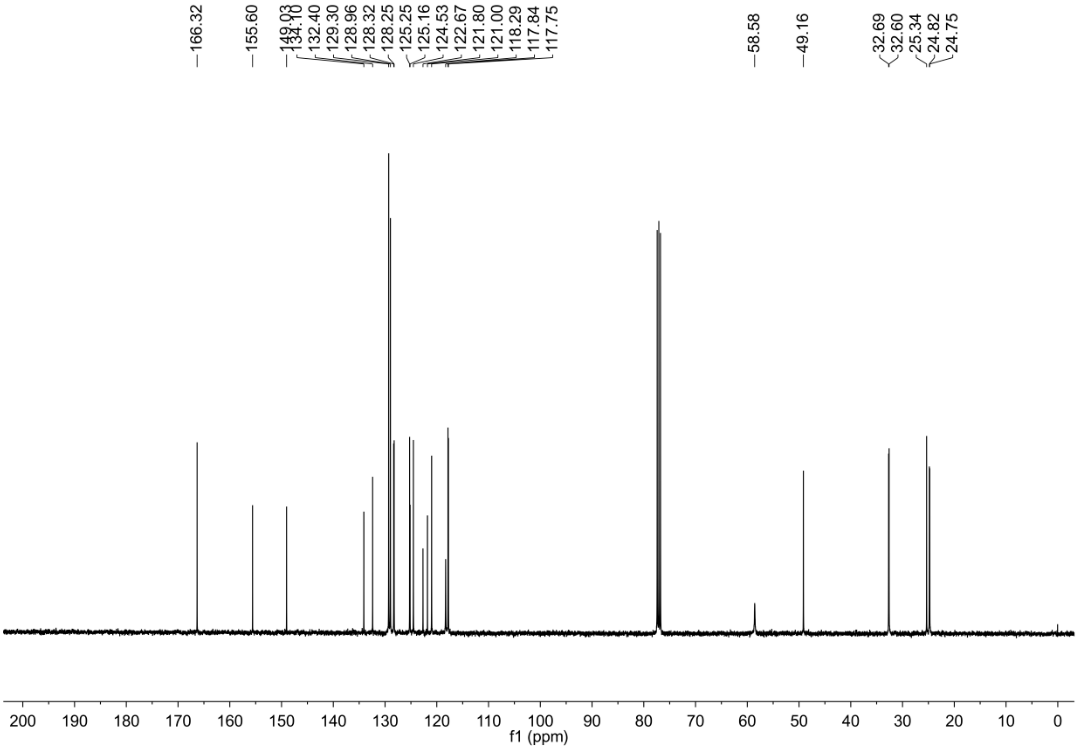

^{1}H NMR spectrum of **6l (**400 MHz, CDCl3**)**

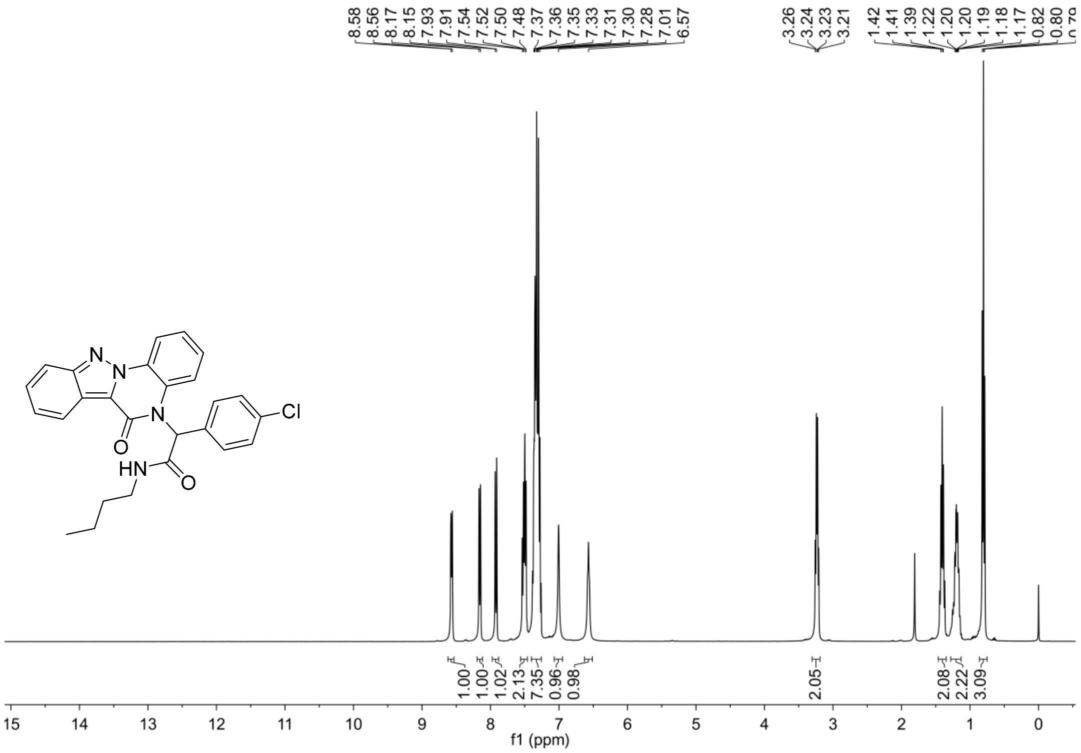

^{13}C NMR spectrum of **6l (**101 MHz, CDCl3**)**

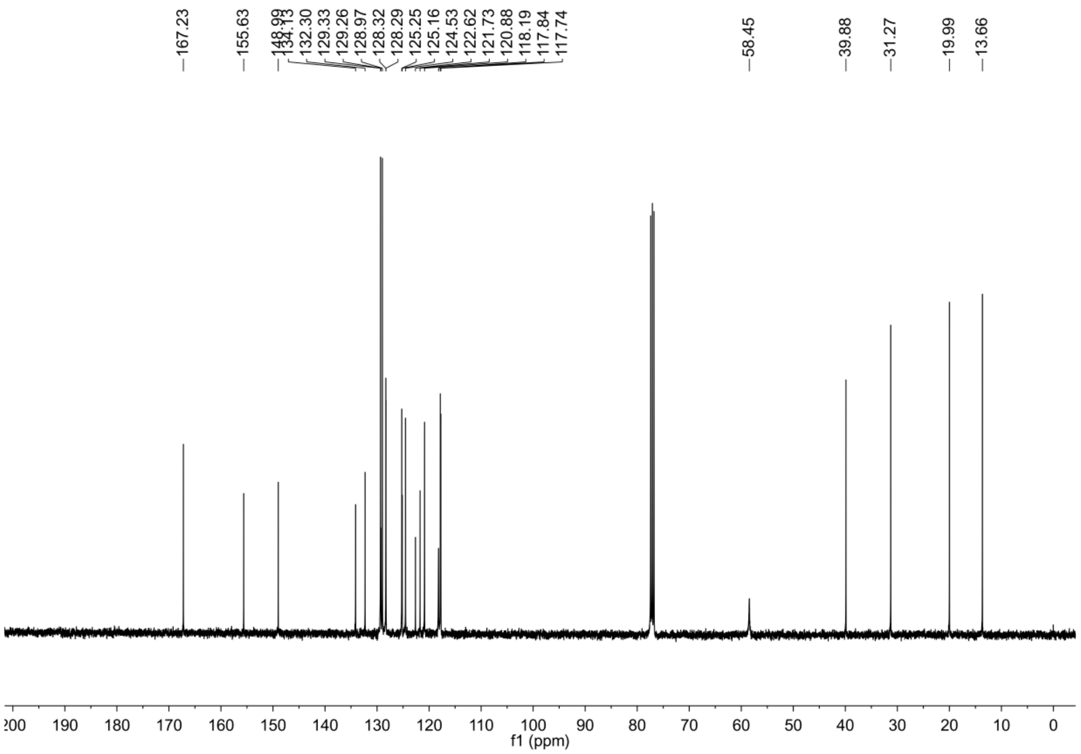

^{1}H NMR spectrum of **6m (**400 MHz, $CDCl_3$**)**

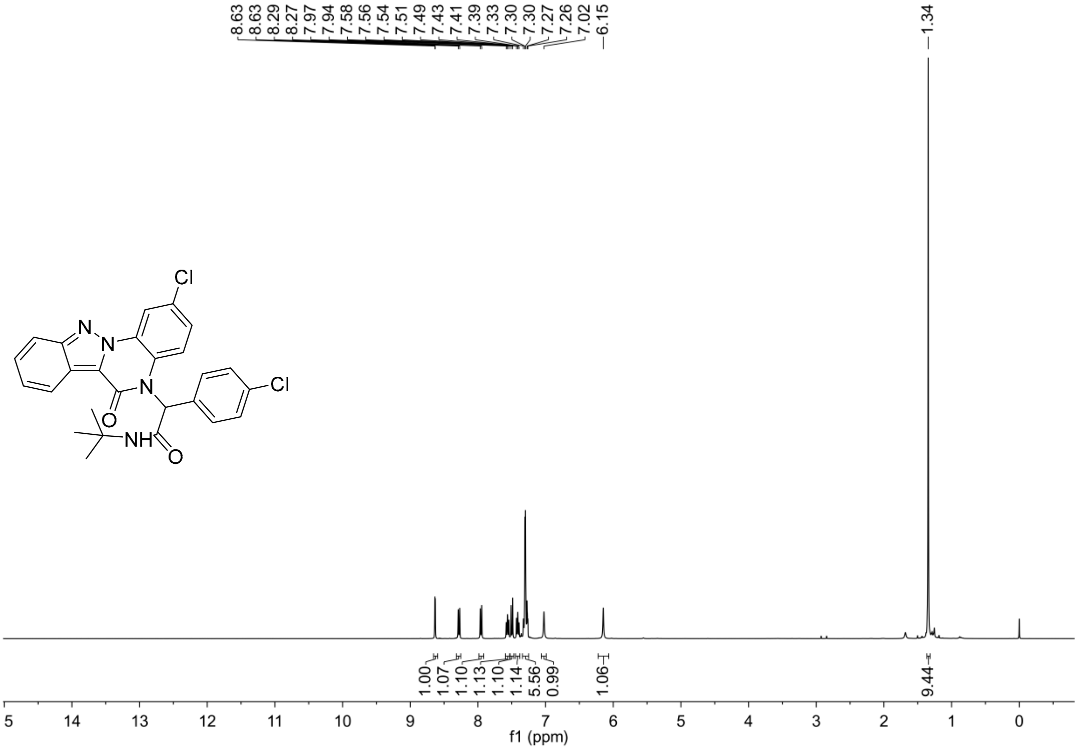

^{13}C NMR spectrum of **6m (**101 MHz, $CDCl_3$**)**

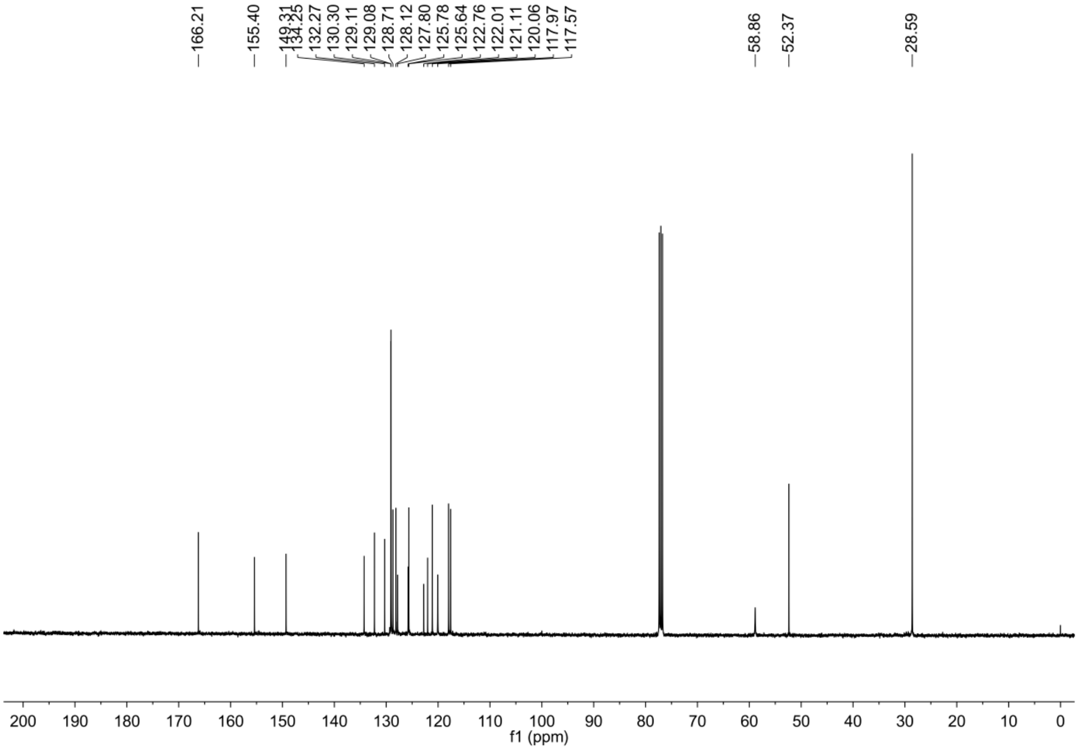

^{1}H NMR spectrum of **6n** (400 MHz, $CDCl_3$)

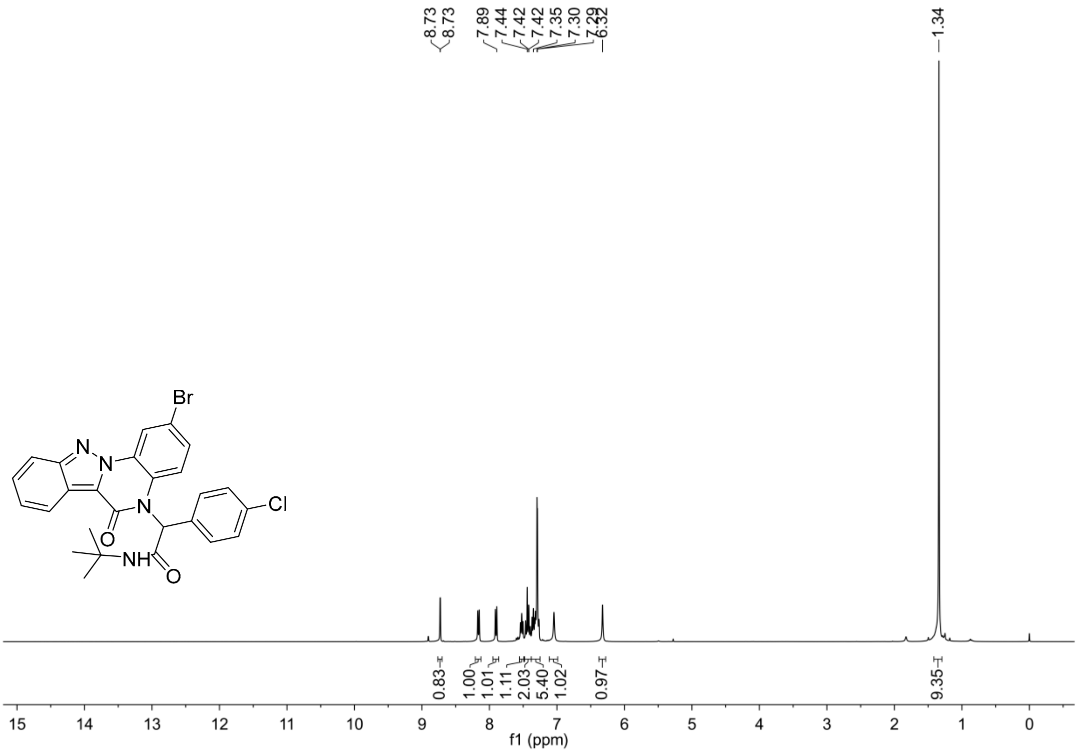

^{13}C NMR spectrum of **6n** (101 MHz, $CDCl_3$)

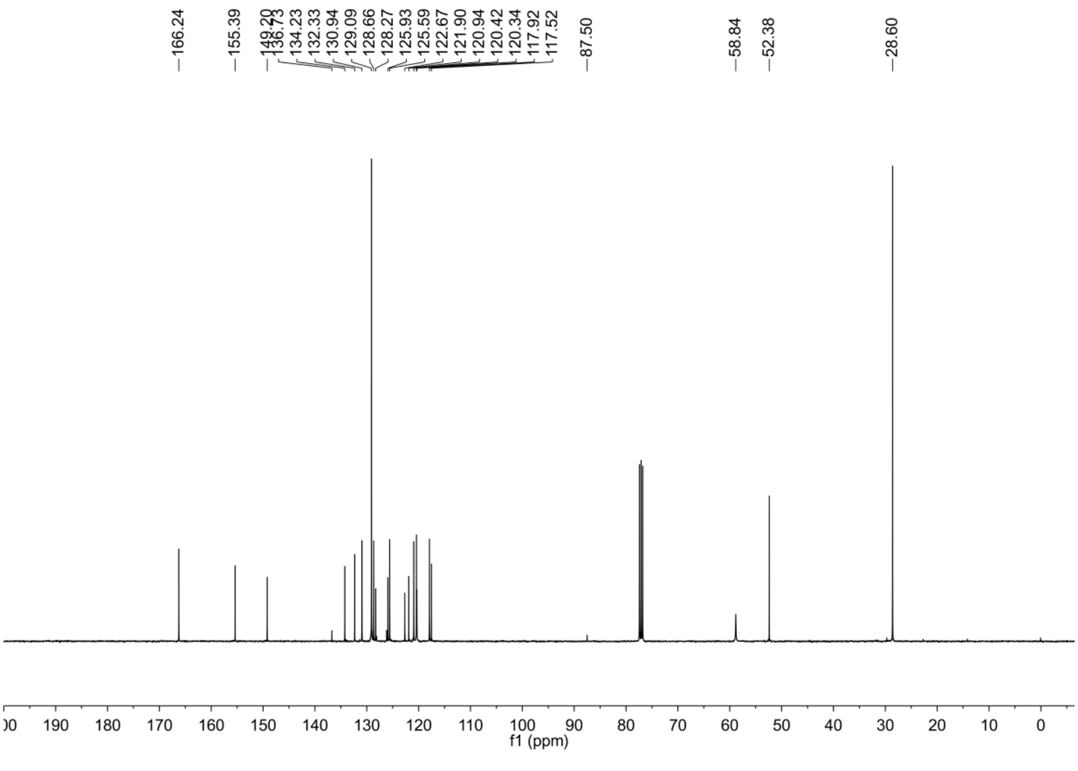

^{1}H NMR spectrum of **6o (**400 MHz, $CDCl_3$**)**

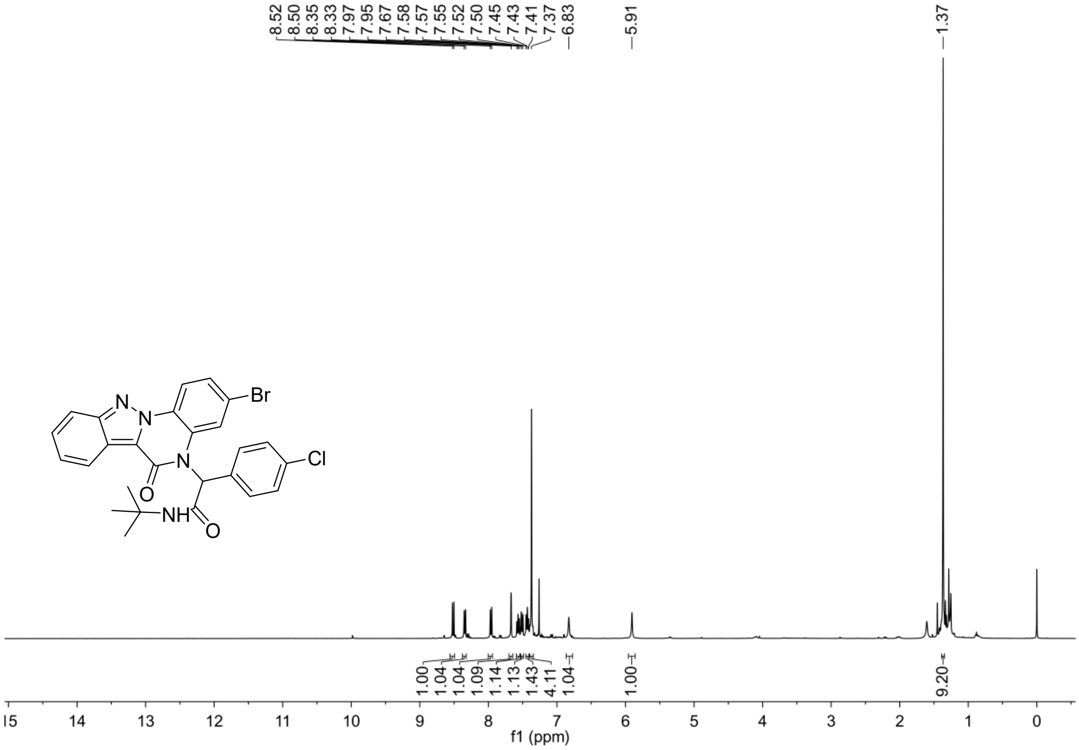

^{13}C NMR spectrum of **6o (**101 MHz, $CDCl_3$**)**

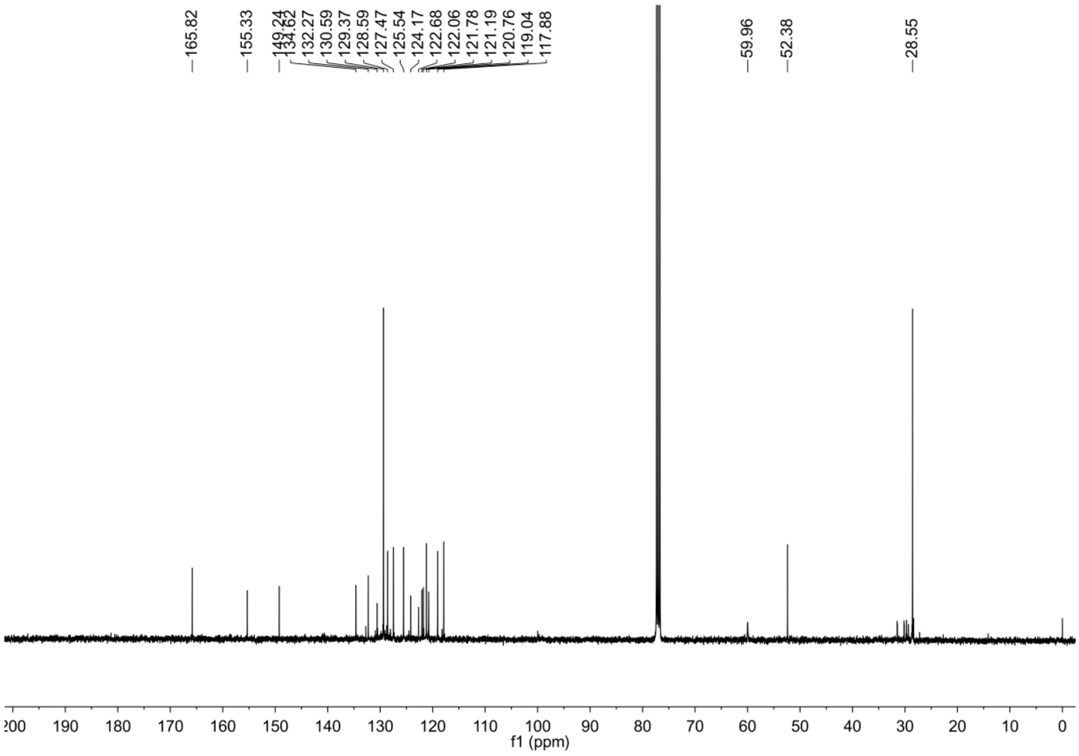

^{1}H NMR spectrum of **6p (**400 MHz, $CDCl_3$**)**

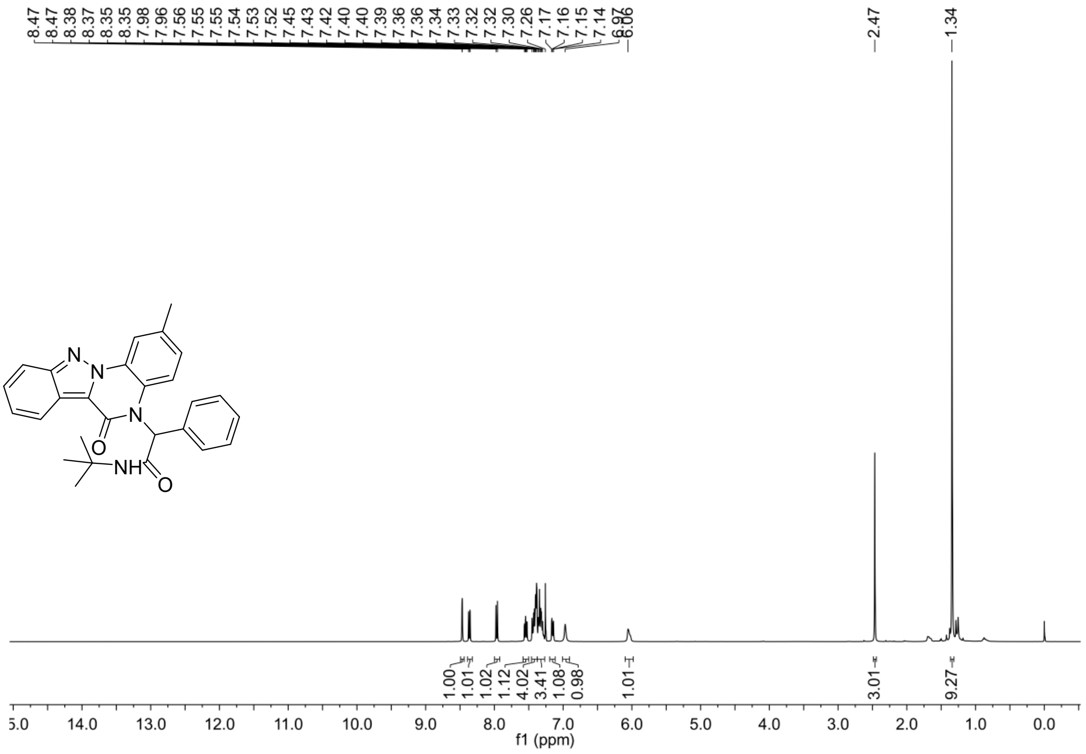

^{13}C NMR spectrum of **6p (**101 MHz, $CDCl_3$**)**

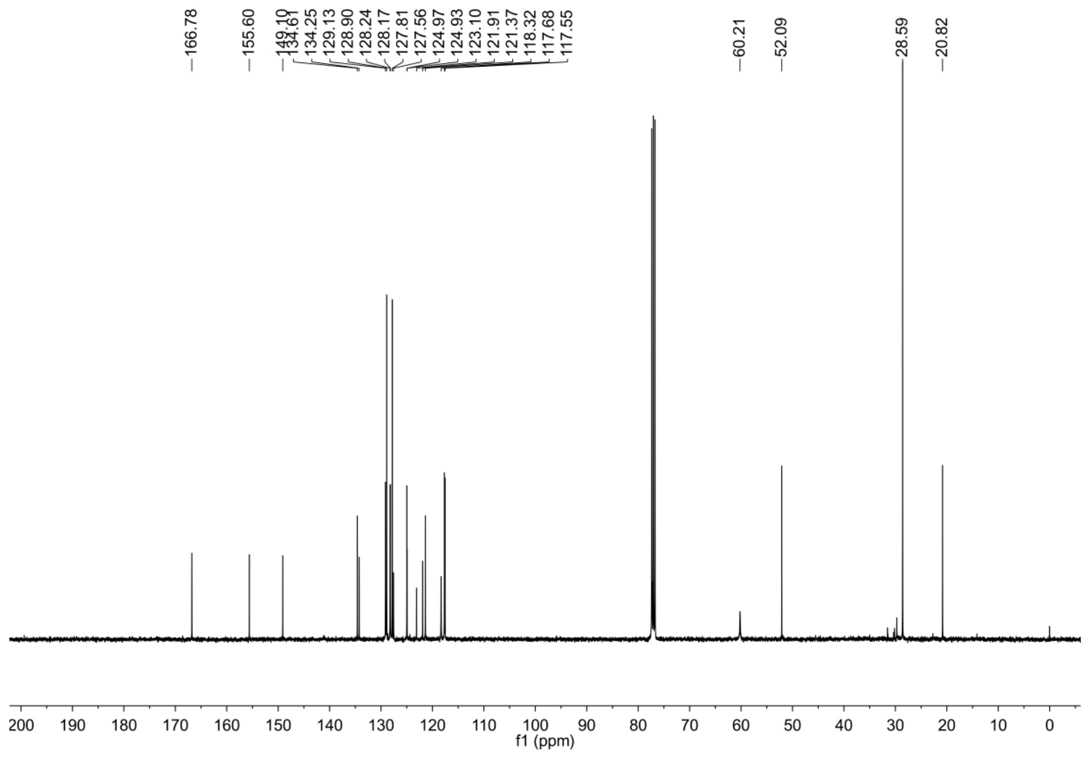

^{1}H NMR spectrum of **6q (**400 MHz, $CDCl_3$**)**

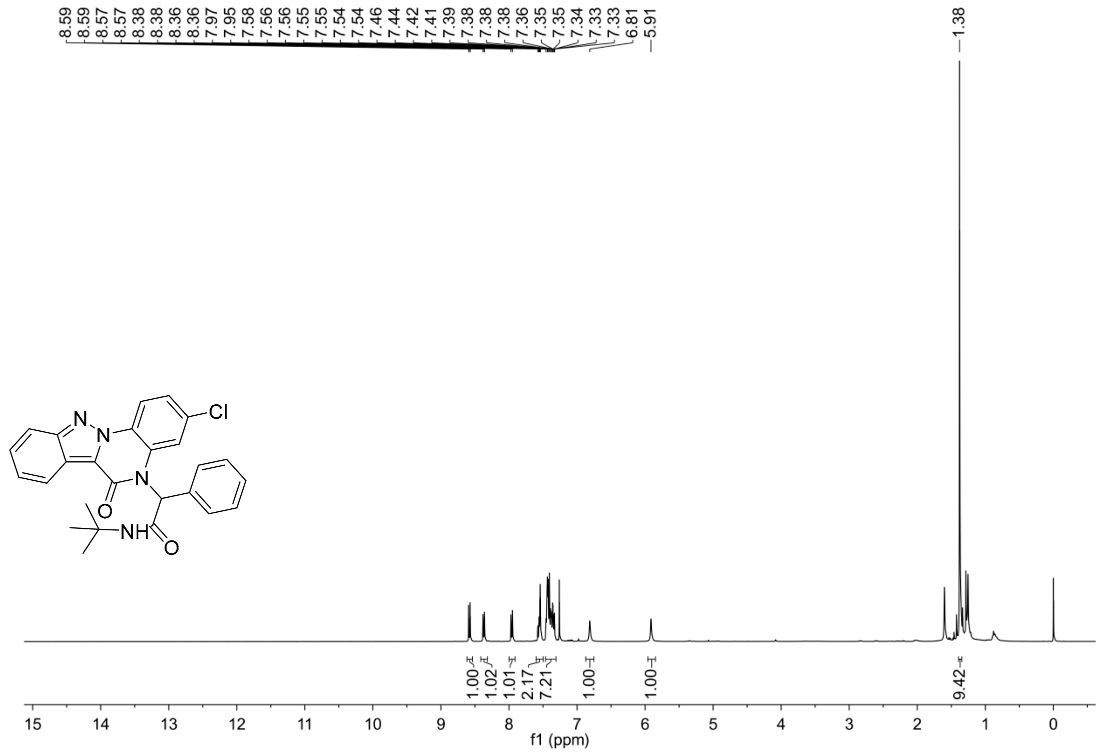

^{13}C NMR spectrum of **6q (**101 MHz, $CDCl_3$**)**

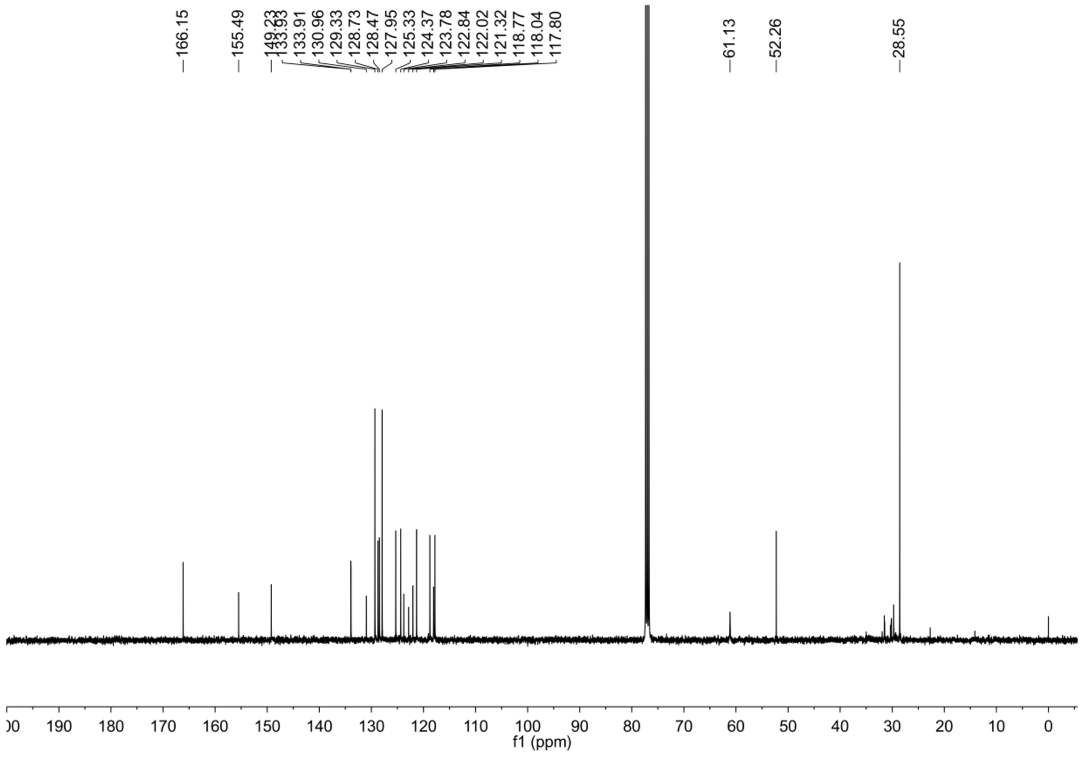

^1H NMR spectrum of **6r (**400 MHz, $CDCl_3$**)**

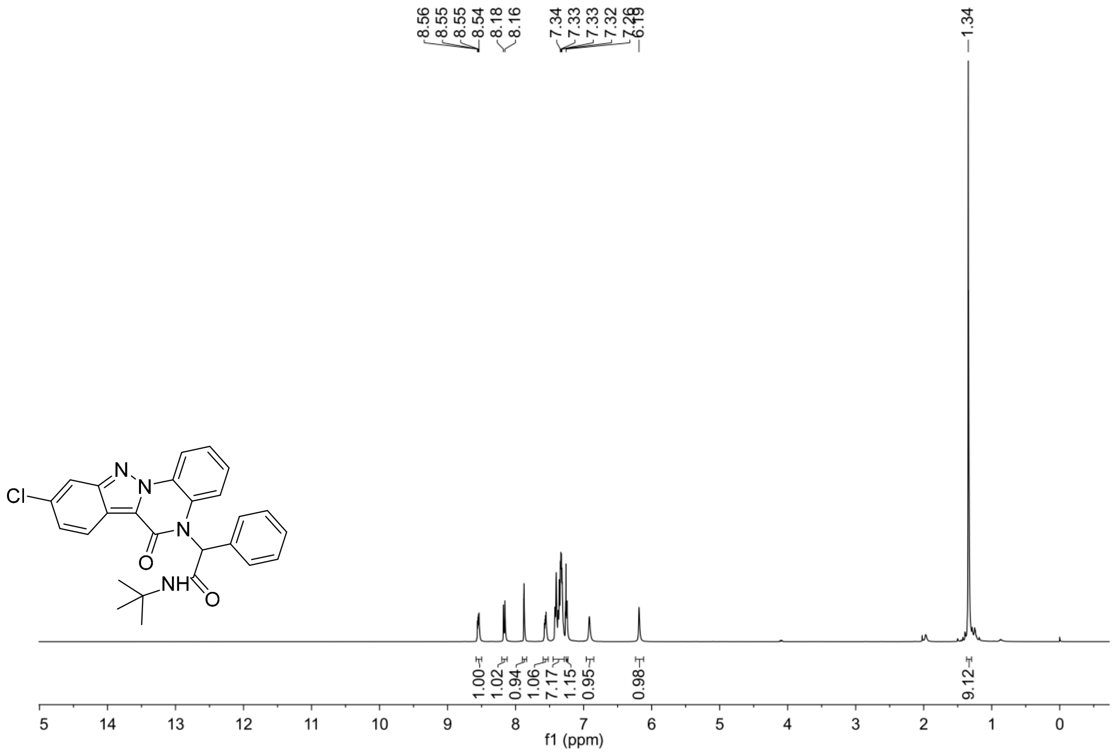

^{13}C NMR spectrum of **6r (**101 MHz, $CDCl_3$**)**

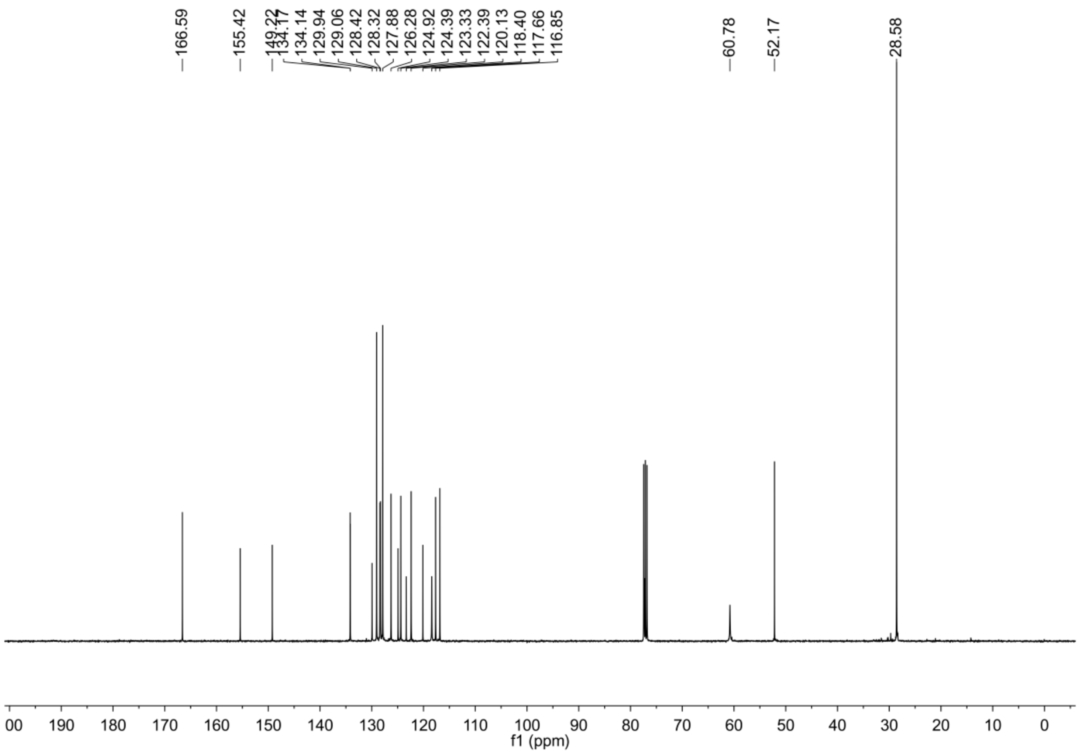

^{1}H NMR spectrum of **6s** (400 MHz, $CDCl_3$)

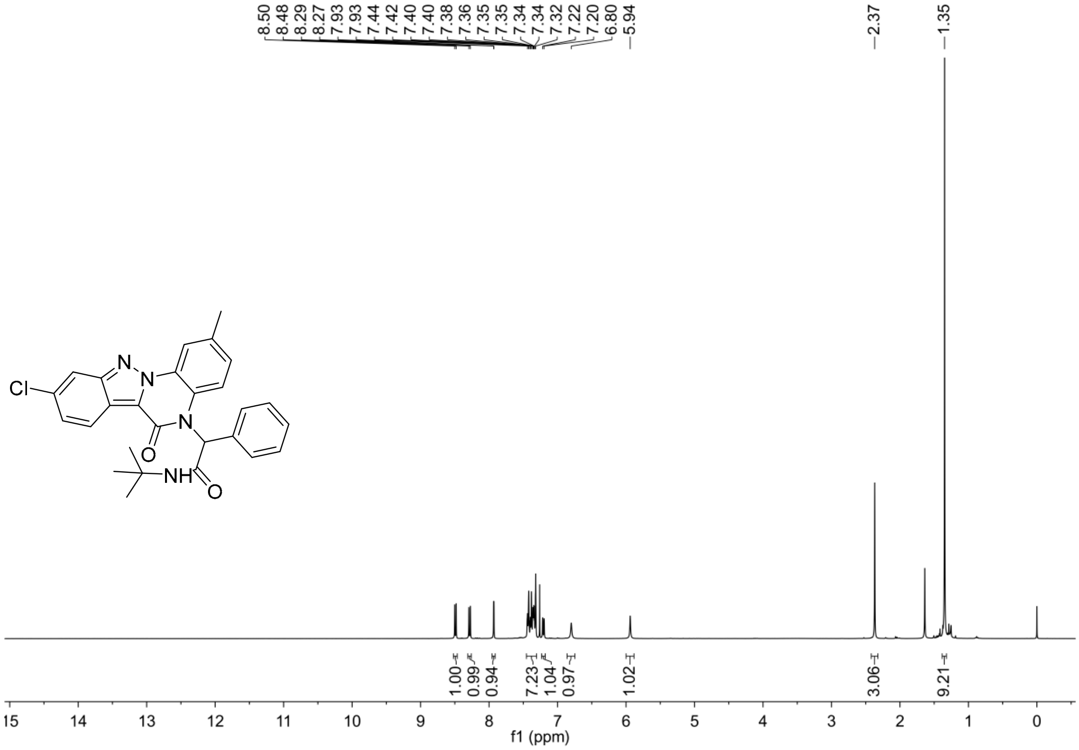

^{13}C NMR spectrum of **6s** (101 MHz, $CDCl_3$)

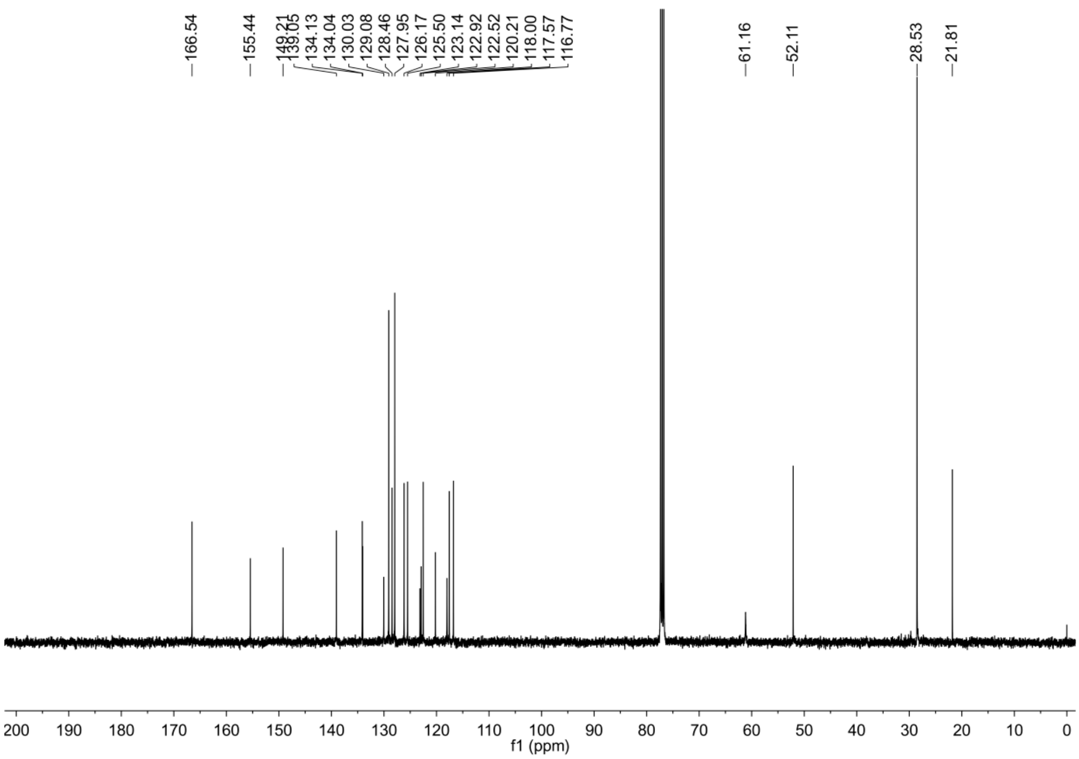

^{1}H NMR spectrum of **6t (**400 MHz, CDCl$_3$**)**

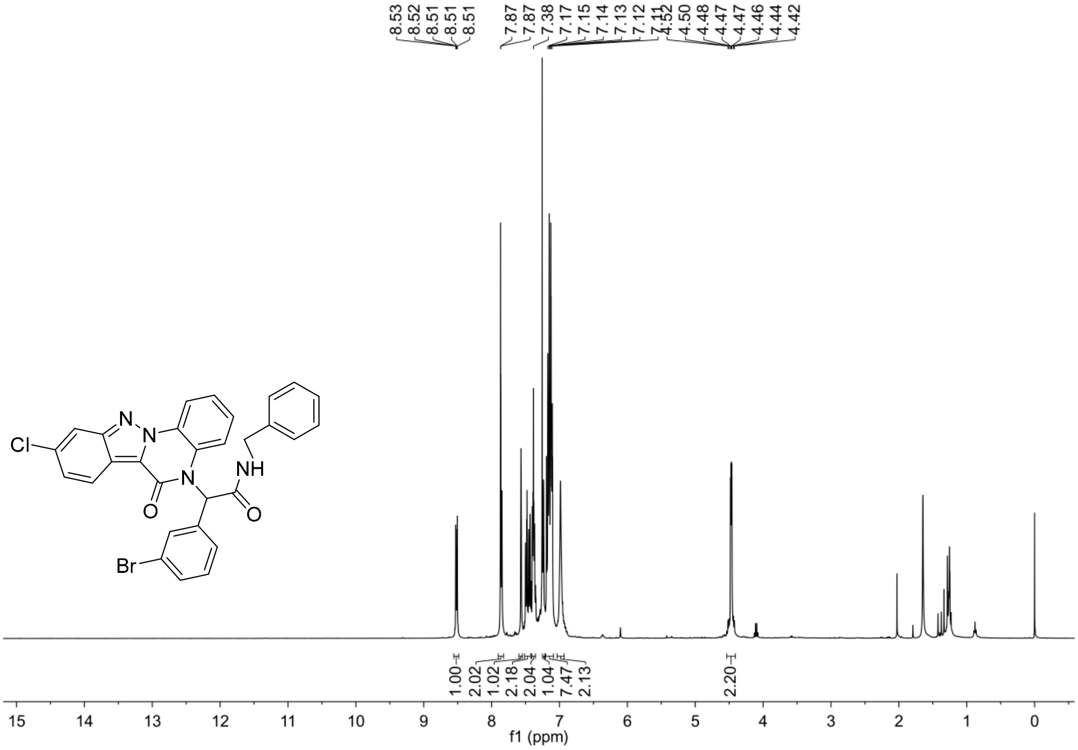

^{13} C NMR spectrum of **6t (**101 MHz, CDCl$_3$**)**

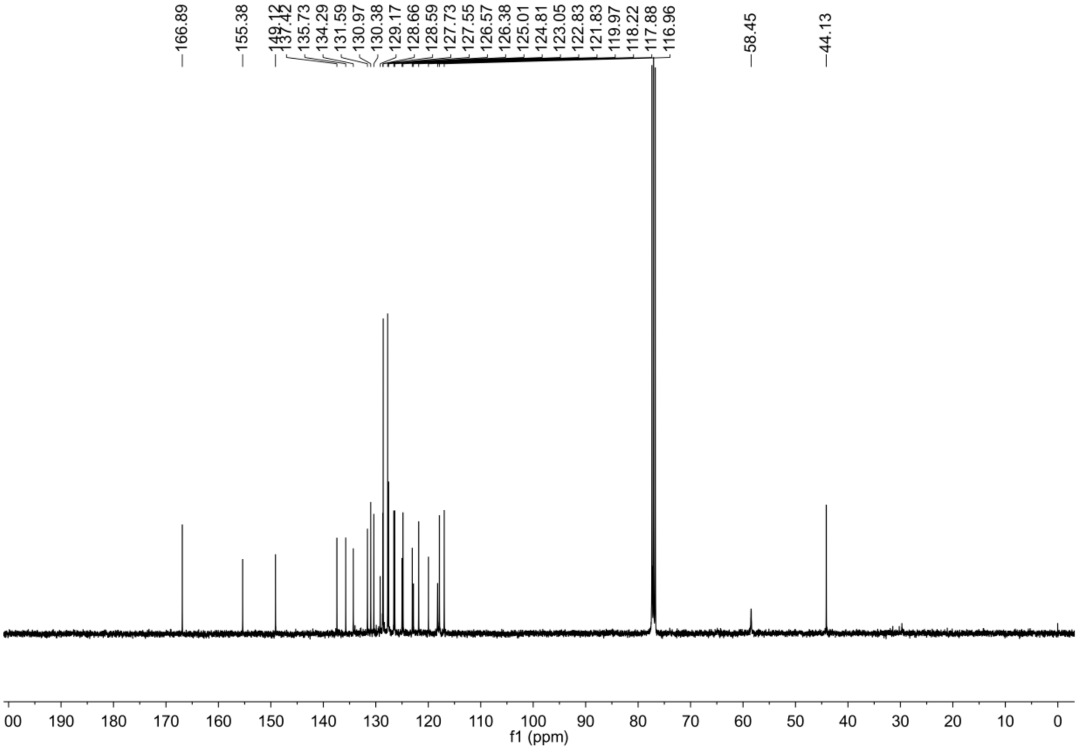

^{1}H NMR spectrum of **6u** (400 MHz, $CDCl_3$)

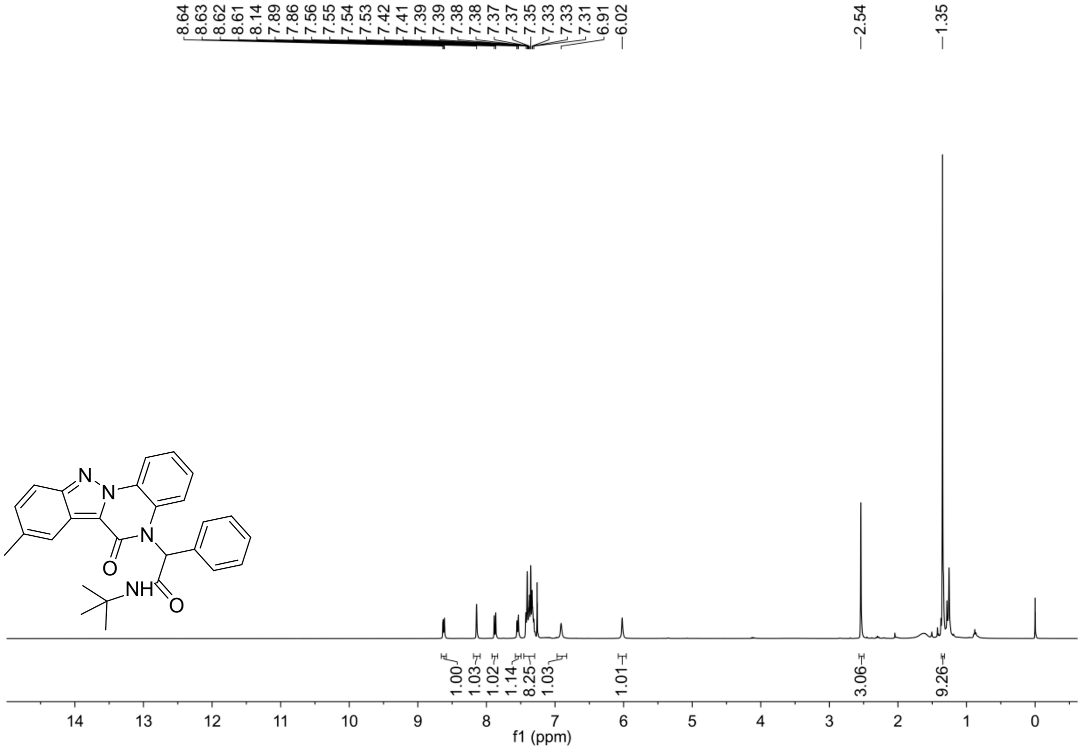

[13] C NMR spectrum of **6u** (101 MHz, $CDCl_3$)

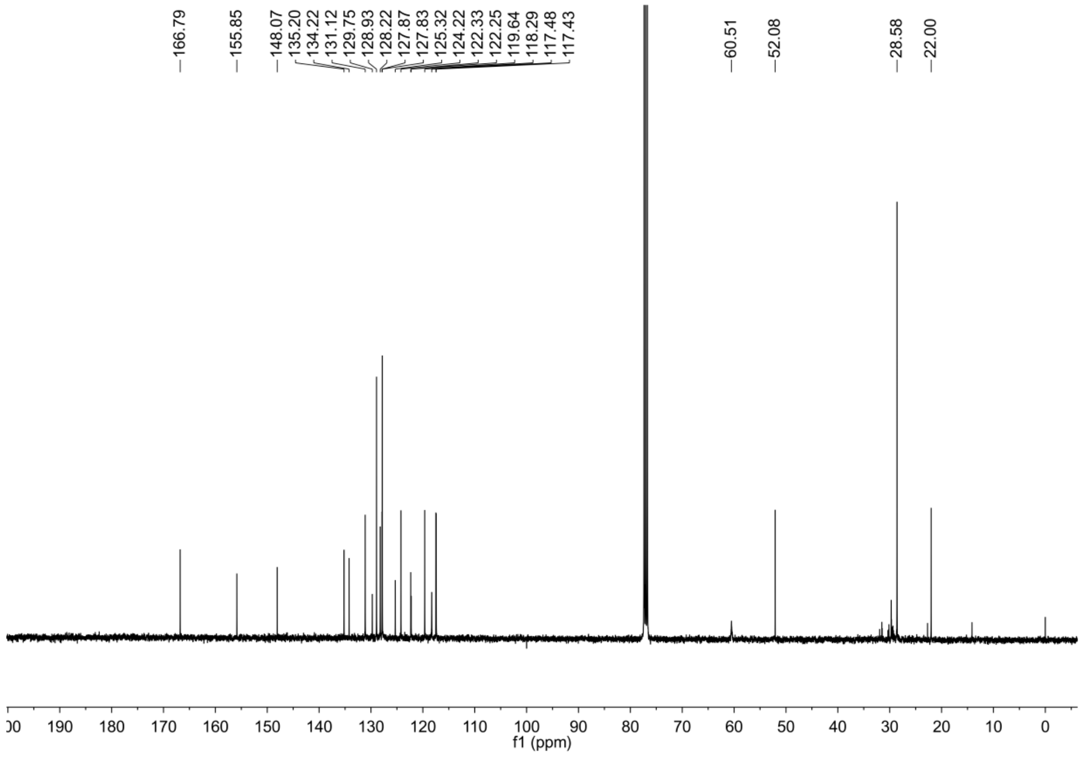

^{1}H NMR spectrum of **6v** (400 MHz, DMSO-*d6*)

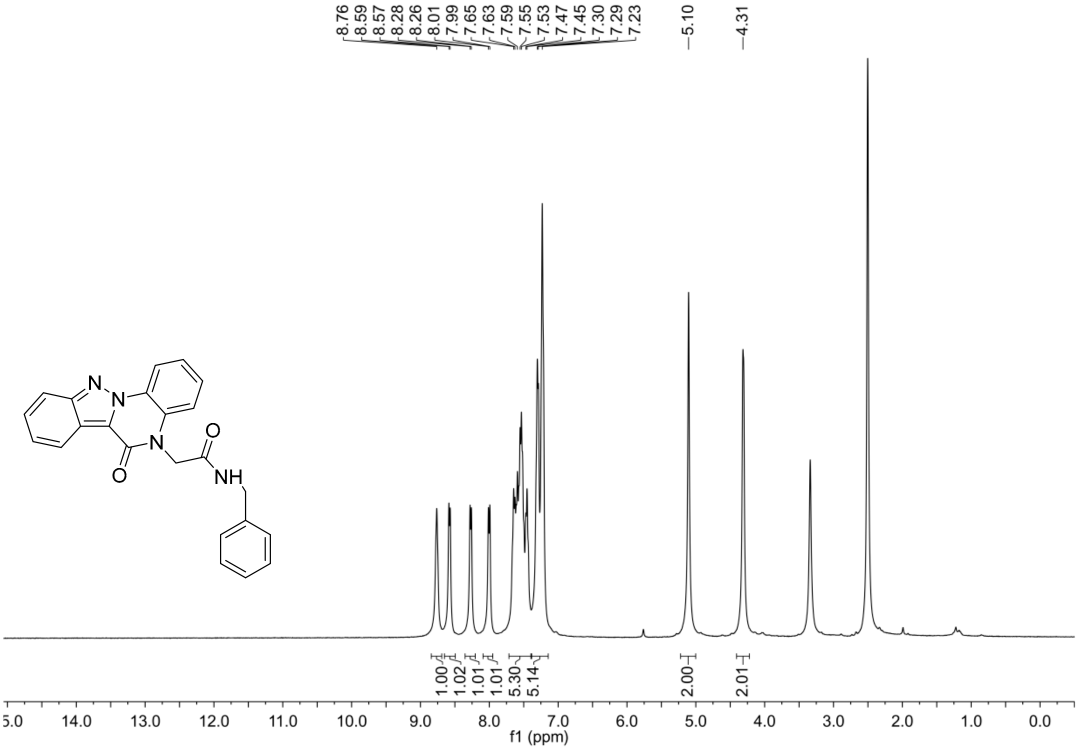

^{13}C NMR spectrum of **6v** (101 MHz, DMSO-*d6*)

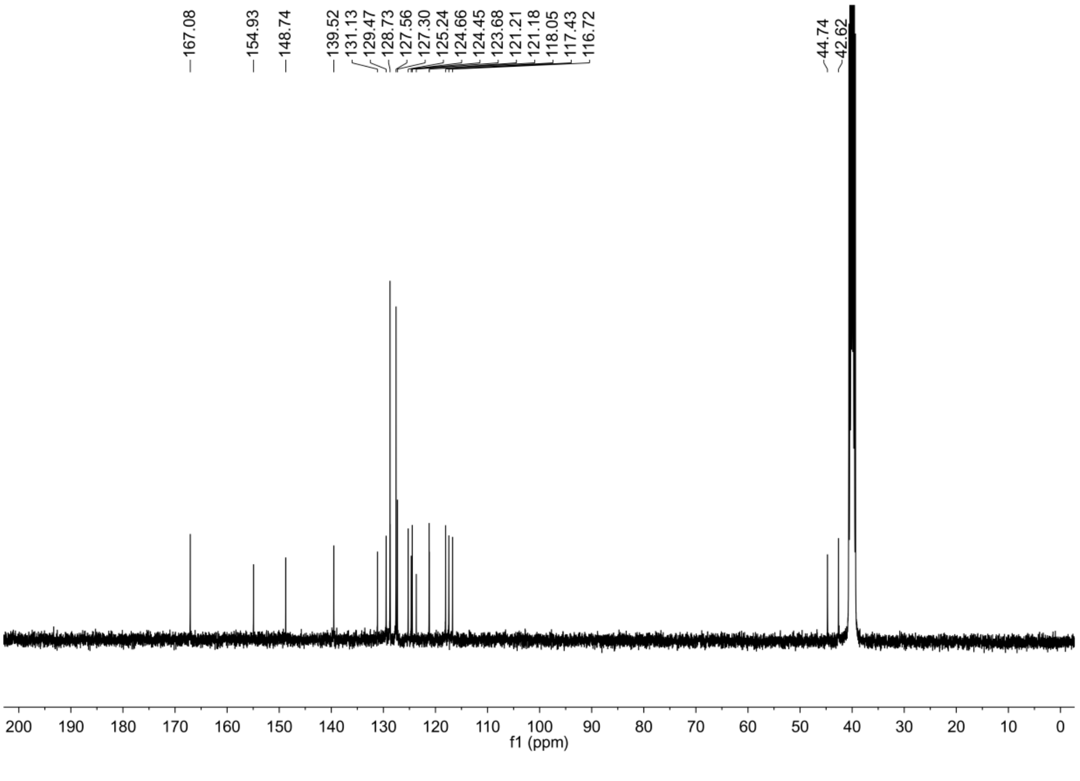

Supplement: Supplementary file 1 [file molecules-29-00464-s001.zip › molecules-2786104-supplementary/molecules-2786104-supplementary.pdf]
